# A One-Pot Approach to Pyridyl Isothiocyanates from Amines

**DOI:** 10.3390/molecules190913631

**Published:** 2014-09-02

**Authors:** Hao Zhang, Rui-Quan Liu, Ke-Chang Liu, Qi-Bo Li, Qing-Yang Li, Shang-Zhong Liu

**Affiliations:** Department of Applied Chemistry, College of Science, China Agricultural University, No. 2 Yuanmingyuan West Road, Beijing 100193, China; E-Mails: cauzhanghao@cau.edu.cn (H.Z.); liuruiquan@cau.edu.cn (R.-Q.L.); lkc@cau.edu.cn (K.-C.L.); lqb@cau.edu.cn (Q.-B.L.); liqingyang@cau.edu.cn (Q.-Y.L.)

**Keywords:** isothiocyanates, bases, iron(III) chloride, pyridyl amines, one-pot process

## Abstract

A one-pot preparation of pyridyl isothiocyanates (ITCs) from their corresponding amines has been developed. This method involves aqueous iron(III) chloride-mediated desulfurization of a dithiocarbamate salt that is generated *in situ* by treatment of an amine with carbon disulfide in the present of DABCO or sodium hydride. The choice of base is of decisive importance for the formation of the dithiocarbamate salts. This one-pot process works well for a wide range of pyridyl ITCs. Utilizing this protocol, some highly electron-deficient pyridyl and aryl ITCs are obtained in moderate to good yields.

## 1. Introduction

Isothiocyanates (ITCs) constitute an important class of natural products that are abundant in many cruciferous vegetables [1]. ITCs have versatile biological activities, ranging from anticancer and chemoprotective properties [2,3,4] to agrochemical activities [5,6,7], and they are also useful intermediates for the synthesis of various sulfur- and nitrogen-containing organic compounds [8], especially for heterocycles [9,10,11,12].

Numerous methods for preparing ITCs have been developed using different starting materials such as amines [13,14,15,16,17,18,19], tertiary alcohols [20], halides [21,22], nitrile oxides [23], azides [5], isocyanides [24,25]. Among these starting materials, amines are usually employed because of their broad availability and versatility. Most reported methods are highly effective for the synthesis of alkyl and electron-rich aryl ITCs, but their applicability to pyridyl-substituted ITCs is limited due to the lower nucleophilicity of pyridyl amines. In fact, the synthesis of ITCs from pyridyl amines proved to be more difficult than that from aryl amines.

**Scheme 1:**
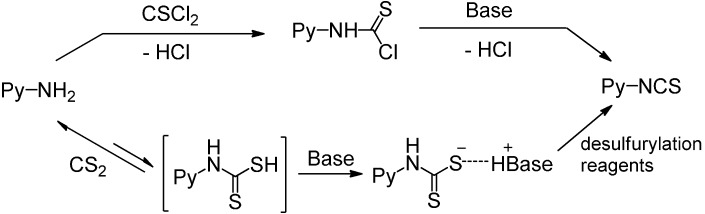
Methods for conversion of amines to pyridyl ITCs.

There are two main methods to convert substituted aminopyridines into the corresponding ITC analogue (Scheme 1). The most well-known method is based on thiophosgene [9], and later refinements of ‘thiocarbonyl transfer’ reagents such as thiocarbonyl-diimidazole [26] and dipyridyl-thionocarbonate [27]. The high toxicity and incompatability of thiophosgene with many functional groups limit its general use, furthermore, these ‘thiocarbonyl transfer’ reagents are not readily available and often do not work as desired due to the formation of thiourea byproducts. Another two-step approach, based on reagent-promoted decomposition of dithiocarbamate salts into ITCs, was first reported by Le Count [28] in 1977. The intermediate dithiocarbamate salts are generated by treatment of amines with carbon disulfide and Et_3_N. Although some desulfurylating reagents for this approach were developed [17,28], the first step, preparing the N-pyridyldithiocarbamate salts, was often neglected. Most of these methods are efficient only for electron-rich pyridyl ITCs, because electron-deficient aminopyridines lack enough reactivity to form dithiocarbamate salts, which results in low yield or excess (hundredfold) use of carbon disulfide. Thus, so far few efficient and general methods have been reported for the preparation of pyridyl ITCs, especially for those with highly electron-withdrawing groups. Therefore, research into an improved method for pyridyl ITCs, which can be used for a broad range of substituents, remains a topic of considerable interest.

## 2. Results and Discussion

In Le Count’s work, iron(III) chloride has been proved to be effective for the decomposition of dithiocarbamate salts, but the preparation of N-pyridyldithiocarbamate salts was seldom investigated, so it became crucial for us to improve their preparation, because once the dithicarbamates were obtained, the desulfurylation step proceeded smoothly [13,16]. In the initial study, 3-amino-6-chloropyridine (**1g**) was chosen as a model substrate to prepare ITCs in a one-pot process (Table 1). At first, the effect of various bases was evaluated by performing the model reaction in tetrahydrofuran (entries 1–9). When inorganic bases (K_2_CO_3_, KOH) and organic bases like 1,8-bis(dimethylamino)naphthalene (Proton Sponge™) or pyridine were employed, the conversion of **1g** was rather low, even after 12 h, giving less than 30% of **4g** (entries 1–4). When triethylamine and potassium *tert*-butoxide was used, the conversion was significantly improved after 12 h (entries 5–6), however, a large amount of thiourea was formed in the case of *t*-BuOK. To our delight, when 1,8-diazabicyclo[5.4.0]-undec-7-ene (DBU), 4-dimethylaminopyridine (DMAP) or 1,4-diazabicyclo[2.2.2]octane (DABCO) were used as base, the conversion was complete within 4 h and **4g** was obtained in excellent yield (entries 7–9). The results was summarized in Table 1 and could not be explained by the strength of the base (p*K*_a_), for example, the substrate **1g** reacted with CS_2_ in the presence of DABCO (p*K*_a_ 8.7) and Et_3_N (p*K*_a_ 10.7), but it did not in the presence of pyridine (p*K*_a_ 5.4) and Proton Sponge™ (p*K*_a_ 12.1). The p*K*_a_ values for protonated base are determined in polar solvents (water, MeCN, DMSO), in which they are dissociated as free ions [29]. However, THF is a nonpolar solvent and has a low dielectric constant, thus, the corresponding ammonium salts in nonpolar solvents are present entirely as ion pairs rather than free ions. To measure ion pairs basicity of some amines in THF, Streitwieser introduced the concept of p*K*_ip_ [30], which refers to the equilibrium between the base and the acid with the H-bonded ion pair, and found that the p*K*_ip_ values are inconsistent with their corresponding p*K*_a_ values [31].

**Table 1:** Optimization of reaction conditions for the synthesis of 2-chloro-5-isothiocyanatopyridine ^a^. 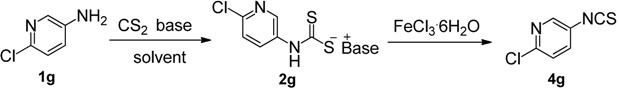

Entry	Solvent	Base	P*K*_a_^b^	P*K*_ip_^c^	Conversion of 1g (%)	Overall Yield (%)
1	THF	K_2_CO_3_	10.3		31	11
2	THF	KOH	15.7		46	25
3	THF	pyridine	5.4	2.2	0	trace
4	THF	Proton sponge	12.1		0	trace
5	THF	Et_3_N	10.7	2.1	85	77
6	THF	*t*-BuOK	29.0		78	trace
7	THF	DABCO	8.7	0.8	100	96
8	THF	DBU	11.6	−3.8	100	90
9	THF	DMAP	9.9	0.61	100	90
10	DMF	DABCO			95	87
11	acetone	DABCO			86	70
12	MeCN	DABCO			84	70
13	EtOH	DABCO			0	trace
14	CH_2_Cl_2_	DABCO			60	48

Notes: ^a^ Reaction conditions: **1g** (1 equiv), CS_2_ (3 equiv), base (2 equiv), solvent, r.t.; FeCl_3_·6H_2_O (2 equiv), r.t., 1 h; ^b^ The dissociation constant of the protonated base in water. Values were collected from refs [32,33]; ^c^ The equilibrium between the base and acidic indicator hydrocarbons InH with the H-bonded ion pairs. p*K*_ip_ = −log*K*_ip_ [30].

A possible mechanism for the formation of pyridyl dithiocarbamate salts is proposed in Scheme 1. The first step, the attack of amine on carbon disulfide to form dithiocarbamic acid, is likely reversible. The driving force of the reaction is most likely the reaction of the dithiocarbamic acid with base to generate the stable dithiocarbamate salts. A greater ion pair basicity corresponds to a tighter ion pair, which facilitates the generation of dithiocarbamates, the ion pair basicities of Et_3_N (p*K*_ip_ 2.1) and DABCO (p*K*_ip_ 0.8) agree with their observed different reactivity. When we used DABCO as the base, an examination of different solvents showed that THF was the best solvent compared with DMF, acetone, MeCN, EtOH, CH_2_Cl_2_ (entries 10–14). Finally, with the optimized conditions for the formation of **2g**, we then found that upon addition of aqueous FeCl_3_ to unpurified **2g** in one-pot, complete conversion to **4g** was observed in about 1 h at room temperature.

Under the reaction conditions outlined above (Table 1, entry 7), the substrate scope of various aminopyridines was examined next (Table 2). The electronic effect of the substituents has a significant influence on the reaction outcome.

**Table 2:** Preparation of aromatic ITCs ^a^. 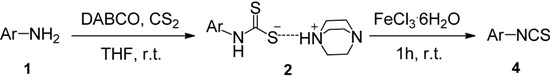

Entry	Amines	Product	CS_2_ (equiv)	Time (h) ^b^	Overall Yield (%)
	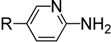	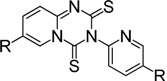			
1	R = H	**4a**	3	4	87
2	R = Me	**4b**	3	4	88
	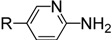	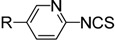			
3	R = F	**4c**	3	12	76
4	R = Cl	**4d**	10	12	81
5	R = Br	**4e**	10	12	83
6	R = CF_3_	**4f**	20	24	42
7	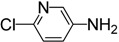	**4g**	3	4	96
8	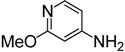	**4h**	3	2	91
9	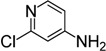	**4i**	10	12	73
	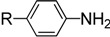	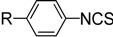			
10	R = CN	**4j**	4	12	87
11	R = NO_2_	**4k**	5	24	77
12	R = CF_3_	**4l**	4	12	85
13	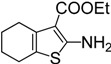	**4m**	4	12	66

Notes: ^a^ Reaction conditions: **1** (8.0 mmol), CS_2_ (excess), DABCO (16.0 mmol), THF (10 mL), r.t.; FeCl_3_·6H_2_O (16.0 mmol), r.t., 1 h; ^b^ The reaction time for the first step.

For example, aminopyridines containing electron donating groups (Me, OMe) afforded good yields of 87%–91% in a relatively short reaction time (entries 2 and 8). Incidently, the corresponding ITCs from 2-aminopyridine and 2-amino-5-methylpyridine have been obtained as dimers, and such dimers slowly dissociate to monomers in hot organic solvent [34,35]. When the 2- or 4-aminopyridines contained halides (entries 3–5, 9), longer reaction times and more equivalents of CS_2_ were required to access **2**, but the corresponding ITCs were still obtained in moderate to good yields, ranging from 73% to 83%. Meanwhile, the position of the amino group on the pyridine also exerted an influence on the reaction outcome; for example, the overall yield of C_6_H_3_ClN_2_S varies for 2-(3-or 4-)aminopyridines (entries 4, 7, 9), and a greater yield was obtained when the amino group is at the *meta* position with respect to the nitrogen atom in the pyridine (96%, entry 7). To our delight, several anilines with strong electron-withdrawing groups, such as NO_2_, CN, and CF_3_ (entries 10–12), were also smoothly converted into the desired ITCs in 77%–87% yields. The approach also worked well for the five-membered heterocyclic substrate (entry 13). However, the desired ITCs could not be detected when highly electron-deficient aminopyridines (such as those with NO_2_, CN, CO_2_Me substituents) were used. Only 5-trifluoromethylpyridyl-2-amine afforded the corresponding ITC in a low yield (42%, entry 6), even after prolonged reaction time and with excess CS_2_. For halide substituents in the *ortho* position of the amino group, no corresponding ITCs were observed. Thus, additional investigations are necessary to develop methods for the preparation of some highly electron-deficient pyridyl ITCs.

**Table 3:** Preparation of highly electron-deficient pyridyl ITCs ^a^. 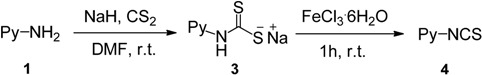

Entry	Amine	Product	Overall Yield (%)
	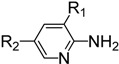	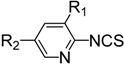	
1	R_1_ = H, R_2_ = CN	**4n**	51
2	R_1_ = H, R_2_ = NO_2_	**4o**	31
3	R_1_ = H, R_2_ = CO_2_Me	**4p**	63
4	R_1_ = Cl, R_2_ = Cl	**4q**	77
5	R_1_ = Cl, R_2_ = H	**4r**	84
6	R_1_ = F, R_2_ = H	**4s**	72
7	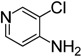	**4t**	49

Notes: ^a^ Reaction conditions: **1** (8.0 mmol), CS_2_ (32.0 mmol), NaH (9.6 mmol), DMF (8 mL), r.t., 6 h; Et_3_N (8.0 mmol), FeCl_3_·6H_2_O (16.0 mmol), r.t., 1 h.

The observed deficiencies in the synthesis of highly electron-deficient pyridyl ITCs inspired us to further optimize the process. The difficulty in the generation of dithiocarbamates is likely due to the weaker nucleophilicity of these amine substrates. In an effort to improve the reactivity, higher reaction temperatures in a variety of solvents were tested. Using methyl 6-aminonicotinate as a test substrate, we found that after 20 h of reflux in THF or DMF, only trace amounts of the corresponding ITCs were observed. We therefore investigated next the use of the strong base NaH to generate the more nucleophilic amide anions prior to CS_2_ addition. After testing various solvents, the use of NaH in DMF was found to be the best choice. The amines was treated with NaH in DMF at 0 °C, then CS_2_ was added, and after 6 h at room temperature, when the amines were fully consumed as monitored by TLC, the reaction mixtures were slowly treated with aqueous FeCl_3_. Using this process, we were able to obtain reasonable yields of several pyridyl ITCs with strong electron-withdrawing groups, such as NO_2_, CN, CO_2_Me, and 3,5-Cl_2_ (Table 3, entries 1–4, 31%–77% yield). This method was also effective for substrates bearing halide substituents in the *ortho* position of the amino moiety (entries 5–7, 49%–84% yield).

## 3. Experimental Section

### 3.1. General Information

Tetrahydrofuran was redistilled in the presence of sodium/benzophenone. Unless otherwise stated, all reagents were commercially available and were used without purification. TLC was performed on pre-coated silica gel glass plates. Flash column chromatography was performed using flash silica gel (200–300 mesh) (Qingdao Haiyang, Qingdao, China). HPLC analyses were performed on an Agilent 1200 Series instrument (Santa Clara, CA, USA, column: Agilent Eclipse XDB-C18, 5 μm, 4.6 × 150 mm). Melting points were determined using a Stuart melting point apparatus and were uncorrected. ^1^H- and ^13^C-NMR spectra were recorded with a 300 MHz spectrometer (Bruker, Fallanden, Switzerland). HRMS and GC-MS were recorded on an Agilent mass spectrometer by the ESI and EI techniques, respectively. All yields given refer to isolated yields.

### 3.2. General Procedure for the Preparation of Isothiocyanates **4a**–**m**

To a solution of amine **1** (8.0 mmol) and DABCO (16 mmol) in anhydrous THF (10 mL) was added dropwise a certain amount of CS_2_. The resulting mixture was stirred at r.t. for several hours until completion by TLC analysis. Then a solution of FeCl_3_·6H_2_O (16 mmol) in water (15 mL) was added rapidly to the well suspended dithiocarbamate **2**, and stirring was continued for 1 h. The aqueous layer was separated and extracted with EtOAc (2 × 10 mL). The combined organic phase was washed with water (2 × 10 mL), and dried over MgSO_4_. After removal of the solvent, the product was purified by flash column chromatography (petroleum ether–EtOAc) to give the corresponding ITCs **4**.

### 3.3. General Procedure for the Preparation of Isothiocyanates **4n**–**t**

To an ice-cold stirred solution of amine **1** (8.0 mmol) in DMF (8 mL) was added NaH (60% in mineral oil; 9.6 mmol) in two portions. After the evolution of gas from the reaction mixture ceased, CS_2_ (32 mmol) was added via syringe pump over about 30 min. The resulting mixture was brought up to r.t. and kept for 6 h, then the mixture was cooled on an ice bath. Et_3_N (8.0 mmol) and a solution of FeCl_3_·6H_2_O (16 mmol) in water (15 mL) were successively added to the dithiocarbamate **3**. After the additions, the mixture was stirred at r.t. for 1 h. The subsequent operations were the same as the workup in the experimental procedure described above.

### 3.4. Characterization Data

*3-(Pyridin-2-yl)-2H-pyrido[1,2-a][1,3,5]triazine-2,4(3H)-dithione* (**4a**) [34]. The crude product purified by column chromatography (petroleum ether/CHCl_3_ = 5:1~1:1, v/v), affording the dimer of 2-pyridyl isothiocyanate as a brick-red solid; yield: 0.95 g (3.48 mmol, 87%); m.p. 110.2–111.1 °C (lit. [28] 112 °C); ^1^H-NMR (CDCl_3_) δ 9.28–9.25 (m, 1H), 8.68–8.66 (m, 1H), 7.95–7.77 (m, 2H), 7.44–7.30 (m, 3H), 7.01–6.96 (m, 1H); ^13^C-NMR (CDCl_3_) δ 179.17 (C=S), 172.26 (C=S), 155.36, 150.34, 146.68, 142.16, 139.03, 132.75, 125.03, 124.14, 123.48, 115.62; HRMS (ESI): *m/z* [M+H]^+^ calcd for C_12_H_9_N_4_S_2_: 273.0269; found: 273.0272.

*7-Methyl-3-(5-methylpyridin-2-yl)-2H-pyrido[1,2-a][1,3,5]triazine-2,4(3H)-dithione* (**4b**) [28]. Brick-red solid, purified by column chromatography (petroleum ether/CHCl_3_ = 5:1~1:1, v/v); yield: 1.06 g (3.52 mmol, 88%); m.p. 137.0–137.4 °C; ^1^H-NMR (CDCl_3_) δ 9.10–9.09 (m, 1H), 8.50 (d, *J* = 2.3 Hz, 1H), 7.78–7.61 (m, 2H), 7.29–7.22 (m, 2H), 2.44 (s, 3H), 2.35 (s, 3H); ^13^C-NMR (CDCl_3_) δ 179.23 (C=S), 172.37 (C=S), 153.31, 150.60, 145.80, 145.07, 139.66, 134.18, 129.95, 126.01, 124.64, 122.71, 18.29, 18.17; HRMS (ESI): *m/z* [M+H]^+^ calcd for C_14_H_13_N_4_S_2_: 301.0582; found: 301.0585.

*5-Fluoro-2-isothiocyanatopyridine* (**4c**). Red solid purified by column chromatography (petroleum ether/EtOAc = 15:1, v/v); yield: 0.94 g (6.08 mmol, 76%); m.p. 21.2–22.4 °C; ^1^H-NMR (CDCl_3_) δ 8.28 (d, *J* = 3.0 Hz, 1H), 7.46 (ddd, *J* = 8.7, 7.3, 3.0 Hz, 1H), 7.12 (dd, *J* = 8.7, 3.9 Hz, 1H); ^13^C-NMR (DMSO-*d*_6_) δ 158.03 (d, ^1^*J*_C-F_ = 253.3Hz), 141.14 (d, ^4^*J*_C-F_ = 2.7Hz), 139.59 (NCS), 137.98 (d, ^2^*J*_C-F_ = 26.3 Hz), 126.71 (d, ^2^*J*_C-F_ = 20.6 Hz), 121.57 (d, ^3^*J*_C-F_ = 5.8 Hz); GC-MS (EI): *m/z* = 154 [M^+^].

*5-Chloro-2-isothiocyanatopyridine* (**4d**). White solid purified by column chromatography (petroleum ether/EtOAc = 15:1, v/v); yield: 1.10 g (6.48 mmol, 81%); m.p. 43.5–44.5 °C (lit. [28] 41–43 °C); ^1^H-NMR (CDCl_3_) δ 8.38 (dd, *J* = 2.6, 0.5 Hz, 1H), 7.68 (dd, *J* = 8.5, 2.6 Hz, 1H), 7.05 (dd, *J* = 8.5, 0.5 Hz, 1H); ^13^C-NMR (CDCl_3_) δ 148.77, 144.61, 142.93 (NCS), 138.32, 130.22, 120.21; HRMS (ESI): *m/z* [M+H]^+^ calcd for C_6_H_4_ClN_2_S: 170.9784; found: 170.9766.

*5-Bromo-2-isothiocyanatopyridine* (**4e**). White solid purified by column chromatography (petroleum ether/EtOAc = 15:1, v/v); yield: 1.42 g (6.64 mmol, 83%); m.p. 73.3–74.2 °C (lit. [28] 74–76 °C); ^1^H-NMR (CDCl_3_) δ 8.48 (d, *J* = 2.5 Hz, 1H), 7.82 (dd, *J* = 8.4, 2.5 Hz, 1H), 7.00 (d, *J* = 8.4 Hz, 1H); ^13^C-NMR (CDCl_3_) δ 151.02, 145.09, 143.00 (NCS), 141.15, 120.69, 118.46; HRMS (ESI): *m/z* [M + H]^+^ calcd for C_6_H_4_BrN_2_S: 214.9279; found: 214.9268.

*2-Isothiocyanato-5-(trifluoromethyl)pyridine* (**4f**) [36]. Red oil purified by column chromatography (petroleum ether/EtOAc = 15:1, v/v) ; yield: 0.69 g (3.36 mmol, 42%); ^1^H-NMR (CDCl_3_) δ 8.73–8.66 (m, 1H), 8.02–7.92 (m, 1H), 7.23–7.20 (m, 1H); ^13^C-NMR (CDCl_3_) δ 149.69, 147.19 (q, ^3^*J*_C-F_ = 4.1 Hz), 144.58 (NCS), 135.95 (q, ^3^*J*_C-F_ = 3.4 Hz), 124.89 (q, ^2^*J*_C-F_ = 33.7 Hz), 122.98 (q, ^1^*J*_C-F_ = 270.7 Hz), 119.07; HRMS (ESI): *m/z* [M+H]^+^ calcd for C_7_H_4_F_3_N_2_S: 205.0047; found: 205.0047.

*2-Chloro-5-isothiocyanatopyridine* (**4g**) [37]. White solid purified by column chromatography (petroleum ether/EtOAc = 20:1, v/v); yield: 1.31 g (7.68 mmol, 96%); m.p. 56.0–57.9 °C; ^1^H-NMR (CDCl_3_) δ 8.31 (dd, *J* = 2.7, 0.7 Hz, 1H), 7.51 (dd, *J* = 8.5, 2.7 Hz, 1H), 7.35 (dd, *J* = 8.5, 0.7 Hz, 1H); ^13^C-NMR (CDCl_3_) δ 148.53, 146.29, 140.14 (NCS), 134.76, 128.41, 124.66; HRMS (ESI): *m/z* [M+H]^+^ calcd for C_6_H_4_ClN_2_S: 170.9784; found: 170.9784.

*4-Isothiocyanato-2-methoxypyridine* (**4h**). White solid purified by column chromatography (petroleum ether/EtOAc = 20:1, v/v); yield: 1.21 g (7.28 mmol, 91%); m.p. 32.4–33.5 °C; ^1^H-NMR (CDCl_3_) δ 8.12 (d, *J* = 5.5 Hz, 1H), 6.71 (dd, *J* = 5.5, 1.8 Hz, 1H), 6.52 (d, *J* = 1.8 Hz, 1H), 3.93 (s, 3H); ^13^C-NMR (CDCl_3_) δ 165.28, 148.18, 141.40, 139.83 (NCS), 113.79, 106.78, 53.75; HRMS (ESI): *m/z* [M+H]^+^ calcd for C_7_H_7_N_2_OS: 167.0279; found: 167.0273.

*2-Chloro-4-isothiocyanatopyridine* (**4i**) [17]. White solid purified by column chromatography (petroleum ether/EtOAc = 15:1, v/v); yield: 0.99 g (5.84 mmol, 73%); m.p. 44.0–44.9 °C, ^1^H-NMR (CDCl_3_) δ 8.37 (dd, *J* = 5.4, 0.6 Hz, 1H), 7.16 (dd, *J* = 1.8, 0.6 Hz, 1H), 7.04 (dd, *J* = 5.4, 1.8 Hz, 1H); ^13^C-NMR (DMSO-*d*_6_) δ 151.47, 151.27, 141.03, 139.32 (NCS), 120.80, 120.11; HRMS (ESI): *m/z* [M+H]^+^ calcd for C_6_H_4_ClN_2_S: 170.9784; found: 170.9806.

*4-Isothiocyanatobenzonitrile* (**4j**). White solid purified by column chromatography (petroleum ether/EtOAc = 20:1, v/v); yield: 1.11 g (6.96 mmol, 87%); m.p. 121.4–122.5 °C (lit. [16] 121–122 °C); ^1^H-NMR (CDCl_3_) δ7.67 (d, *J* = 8.7 Hz, 2H), 7.30 (d, *J* = 8.7 Hz, 1H); ^13^C-NMR (CDCl_3_) δ 139.73 (NCS), 136.05, 133.55, 126.39, 117.79, 110.62; HRMS (ESI): *m/z* [M+H]^+^ calcd for C_8_H_5_N_2_S: 161.0173; found: 161.0158.

*1-Isothiocyanato-4-nitrobenzene* (**4k**). White solid purified by column chromatography (petroleum ether/EtOAc = 20:1, v/v); yield: 1.11 g (6.16 mmol, 77%); m.p. 109.4–110.2 °C (lit. [18] 108–109 °C); ^1^H-NMR (CDCl_3_) δ 8.25 (d, *J* = 9.0 Hz, 2H), 7.36 (d, *J* = 9.0 Hz, 2H); ^13^C-NMR (CDCl_3_) δ 145.80, 140.31 (NCS), 137.90, 126.32, 125.23; HRMS (ESI): *m/z* [M+H]^+^ calcd for C_7_H_5_N_2_O_2_S: 181.0072; found: 181.0054.

*1-Isothiocyanato-4-(trifluoromethyl)benzene* (**4l**). White solid purified by column chromatography (petroleum ether/EtOAc = 20:1, v/v); yield: 1.38 g (6.80 mmol, 85%); m.p. 40.1–41.2 °C (lit. [16] 40–41 °C); ^1^H-NMR (CDCl_3_) δ 7.61 (d, *J* = 8.3 Hz, 2H), 7.30 (d, *J* = 8.3 Hz, 2H); ^13^C-NMR (CDCl_3_) δ 138.47 (NCS), 135.00, 129.05 (q, ^2^*J*_C-F_ = 32.9 Hz), 126.76 (q, ^3^*J*_C-F_ = 3.7 Hz), 125.92, 123.55 (q, ^1^*J*_C-F_ = 270.7 Hz); GC-MS (EI): *m/z* = 203 [M^+^].

*Ethyl 2-isothiocyanato-4,5,6,7-tetrahydrobenzo[b]-thiophene-3-carboxylate* (**4m**). Yellow solid purified by column chromatography (petroleum ether/EtOAc = 10:1, v/v); yield: 1.41 g (5.28 mmol, 66%); m.p. 45.3–45.7 °C (lit. [17] 45–46 °C); ^1^H-NMR (CDCl_3_) δ 4.34 (q, *J* = 7.1 Hz, 2H), 2.77 (t, *J* = 5.7 Hz, 2H), 2.64 (t, *J* = 5.7 Hz, 2H), 1.83–1.76 (m, 4H), 1.40 (t, *J* = 7.1 Hz, 3H); ^13^C-NMR (CDCl_3_) δ 161.89, 137.33 (NCS), 134.72, 132.55, 131.96, 126.49, 60.66, 26.07, 24.88, 22.62, 22.21, 14.35; GC-MS (EI): *m/z* = 267 [M^+^].

*6-Isothiocyanatonicotinonitrile* (**4n**). Yellow solid purified by column chromatography (petroleum ether/EtOAc = 10:1, v/v); yield: 0.66 g (4.08 mmol, 51%); m.p. 68.3–69.5 °C; ^1^H-NMR (CDCl_3_) δ 8.72 (dd, *J* = 2.3, 0.8 Hz, 1H), 7.99 (dd, *J* = 8.3, 2.3 Hz, 1H), 7.18 (dd, *J* = 8.3, 0.8 Hz, 1H); ^13^C-NMR (CDCl_3_) δ 153.14, 149.86, 145.93 (NCS), 141.78, 119.30, 115.85, 107.82; HRMS (ESI): *m/z* [M+H]^+^ calcd for C_7_H_4_N_3_S: 162.0126; found: 162.0109.

*2-Isothiocyanato-5-nitropyridine* (**4o**). Yellow solid purified by column chromatography (petroleum ether/EtOAc = 10:1, v/v); yield: 0.45 g (2.48 mmol, 31%); m.p. 50.3–51.0 °C; ^1^H-NMR (CDCl_3_) δ 9.27 (d, *J* = 2.8 Hz, 1H), 8.51 (dd, *J* = 8.7, 2.8 Hz, 1H), 7.22 (d, *J* = 8.7 Hz, 1H); ^13^C-NMR (CDCl_3_) δ 151.57, 146.46, 146.06, 142.11 (NCS), 134.07, 119.08; HRMS (ESI): *m/z* [M+H]^+^ calcd for C_6_H_4_N_3_O_2_S: 182.0024; found: 182.0019.

*Methyl 6-isothiocyanatonicotinate* (**4p**). White solid purified by column chromatography (petroleum ether/EtOAc = 10:1, v/v); yield: 0.98 g (5.04 mmol, 63%); m.p. 87.2–88.2 °C; ^1^H-NMR (CDCl_3_) δ 9.03 (dd, *J* = 2.3, 0.8 Hz, 1H), 8.31 (dd, *J* = 8.3, 2.3 Hz, 1H), 7.15 (dd, *J* = 8.3, 0.8 Hz, 1H), 3.96 (s, 3H); ^13^C-NMR (CDCl_3_) δ 164.62, 151.51, 149.80, 143.86 (NCS), 139.76, 124.34, 118.96, 52.46; HRMS (ESI): *m/z* [M+H]^+^ calcd for C_8_H_7_N_2_O_2_S: 195.0228; found: 195.0263.

*3,5-Dichloro-2-isothiocyanatopyridine* (**4q**). White solid purified by column chromatography (petroleum ether/EtOAc = 15:1, v/v); yield: 1.26 g (6.16 mmol, 77%); m.p. 51.5–52.6 °C; ^1^H-NMR (CDCl_3_) δ 8.27 (d, *J* = 2.3 Hz, 1H), 7.77 (d, *J* = 2.3 Hz, 1H); ^13^C-NMR (CDCl_3_) δ 146.37, 144.32 (NCS), 142.07, 137.92, 129.92, 127.89; HRMS (ESI): *m/z* [M+H]^+^ calcd for C_6_H_3_Cl_2_N_2_S: 204.9394; found: 204.9374.

*3-Chloro-2-isothiocyanatopyridine* (**4r**). White oil purified by column chromatography (petroleum ether/EtOAc = 10:1, v/v); yield: 1.14 g (6.72 mmol, 84%); ^1^H-NMR (CDCl_3_) δ 8.32 (dd, *J* = 4.7, 1.6 Hz, 1H), 7.78 (dd, *J* = 8.0, 1.6 Hz, 1H), 7.21 (dd, *J* = 8.0, 4.7 Hz, 1H); ^13^C-NMR (CDCl_3_) δ 147.36, 143.36, 142.91 (NCS), 138.37, 127.65, 122.82; HRMS (ESI): *m/z* [M+H]^+^ calcd for C_6_H_4_ClN_2_S: 170.9784; found: 170.9775.

*3-Fluoro-2-isothiocyanatopyridine* (**4s**). Red oil purified by column chromatography (petroleum ether/EtOAc = 10:1, v/v); yield: 0.89 g (5.76 mmol, 72%); ^1^H-NMR (CDCl_3_) δ 8.11–8.09 (m, 1H), 7.46–7.40 (m, 1H), 7.20–7.15 (m, 1H); ^13^C-NMR (CDCl_3_) δ 153.85 (d, ^1^*J*_C-F_ = 262.7 Hz), 144.90 (NCS), 144.40 (d, ^3^*J*_C-F_ = 5.8 Hz), 134.95 (d, ^2^*J*_C-F_ = 13.3 Hz), 124.13 (d, ^2^*J*_C-F_ = 16.8 Hz), 123.19 (d, ^4^*J*_C-F _= 3.0 Hz); HRMS (ESI): *m/z* [M+H]^+^ calcd for C_6_H_4_FN_2_S: 155.0079; found: 155.0051.

*3-Chloro-4-isothiocyanatopyridine* (**4t**). White solid purified by column chromatography (petroleum ether/EtOAc = 10:1, v/v); yield: 0.67 g (3.92 mmol, 49%); m.p. 30.4–31.5 °C; ^1^H-NMR (CDCl_3_) δ 8.62 (d, *J* = 0.5 Hz, 1H), 8.45 (d, *J* = 5.2 Hz, 1H), 7.11 (dd, *J* = 5.2, 0.5 Hz, 1H); ^13^C-NMR (CDCl_3_) δ 150.36, 148.81, 143.11 (NCS), 137.78, 128.99, 120.14; HRMS (ESI): *m/z* [M+H]^+^ calcd for C_6_H_4_ClN_2_S: 170.9784; found: 170.9771.

## 4. Conclusions

In summary, we have developed a facile and environmentally friendly method for the preparation of various pyridyl ITCs from amines via a one-pot process. In comparison to existing methods, our procedure for the synthesis of highly electron-deficient pyridyl ITCs without using dangerous thiophosgene is simple yet efficient. The employed reagents are inexpensive and of low toxicity and the procedure is operationally simple, affording a wide range of pyridyl ITCs in moderate to excellent yields. Based on these characteristics, we envision that this method will be useful to the synthetic community.

## Supplementary Materials

Supplementary materials can be accessed at: http://www.mdpi.com/1420-3049/19/9/13631/s1.

## Supplementary Files

Supplementary File 1
