# Supplementary material for: A One-Pot Approach to Pyridyl Isothiocyanates from Amines
Source: Molecules. 2014 Sep 2;19(9):13631–42. doi: 10.3390/molecules190913631 (PMC6271198; doi:10.3390/molecules190913631)
Supplement: Supplementary File 1 [file molecules-19-13631-s001.pdf]

Supplementary Materials

Table of contents

^{1}H- and ^{13}C-NMR spectra of compound **4a**	S2
^{1}H- and ^{13}C-NMR spectra of compound **4b**	S3
^{1}H- and ^{13}C-NMR spectra of compound **4c**	S4
^{1}H- and ^{13}C-NMR spectra of compound **4d**	S5
^{1}H- and ^{13}C-NMR spectra of compound **4e**	S6
^{1}H- and ^{13}C-NMR spectra of compound **4f**	S7
^{1}H- and ^{13}C-NMR spectra of compound **4g**	S8
^{1}H- and ^{13}C-NMR spectra of compound **4h**	S9
^{1}H- and ^{13}C-NMR spectra of compound **4i**	S10
^{1}H- and ^{13}C-NMR spectra of compound **4j**	S11
^{1}H- and ^{13}C-NMR spectra of compound **4k**	S12
^{1}H- and ^{13}C-NMR spectra of compound **4l**	S13
^{1}H- and ^{13}C-NMR spectra of compound **4m**	S14
^{1}H- and ^{13}C-NMR spectra of compound **4n**	S15
^{1}H- and ^{13}C-NMR spectra of compound **4o**	S16
^{1}H- and ^{13}C-NMR spectra of compound **4p**	S17
^{1}H- and ^{13}C-NMR spectra of compound **4q**	S18
^{1}H- and ^{13}C-NMR spectra of compound **4r**	S19
^{1}H- and ^{13}C-NMR spectra of compound **4s**	S20
^{1}H- and ^{13}C-NMR spectra of compound **4t**	S21

Figure S1. ^{1}H-NMR spectra of compound **4a**.

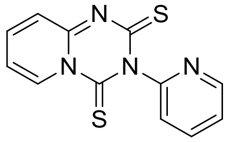

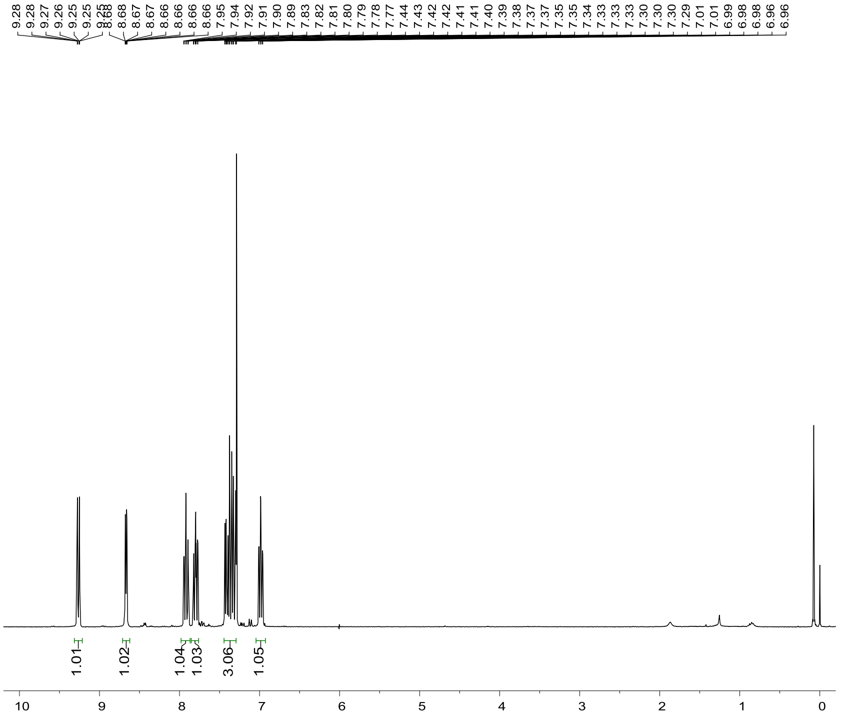

Parameter	Value
Title	zhanghao-H.41.fid
Comment	1H NMR of zh-pydimer
Origin	UXNMR, Bruker Analytische Messtechnik GmbH
Owner	root
Site	
Spectrometer	dpx300
Author	
Solvent	CDCl3
Temperature	24.0
Pulse Sequence	zg30
Experiment	1D
Probe	5 mm PABBO BB-1H Z-GRD Z862701/ 0035
Number of Scans	8
Receiver Gain	124
Relaxation Delay	2.0000
Pulse Width	10.7000
Acquisition Time	5.4658
Acquisition Date	2013-12-18T15:17:00
Modification Date	2013-12-18T15:18:15
Class	
Spectrometer Frequency	300.13
Spectral Width	5995.2
Lowest Frequency	-1195.5
Nucleus	1H
Acquired Size	32768
Spectral Size	65536

Figure S2. ^{13}C-NMR spectra of compound **4a**.

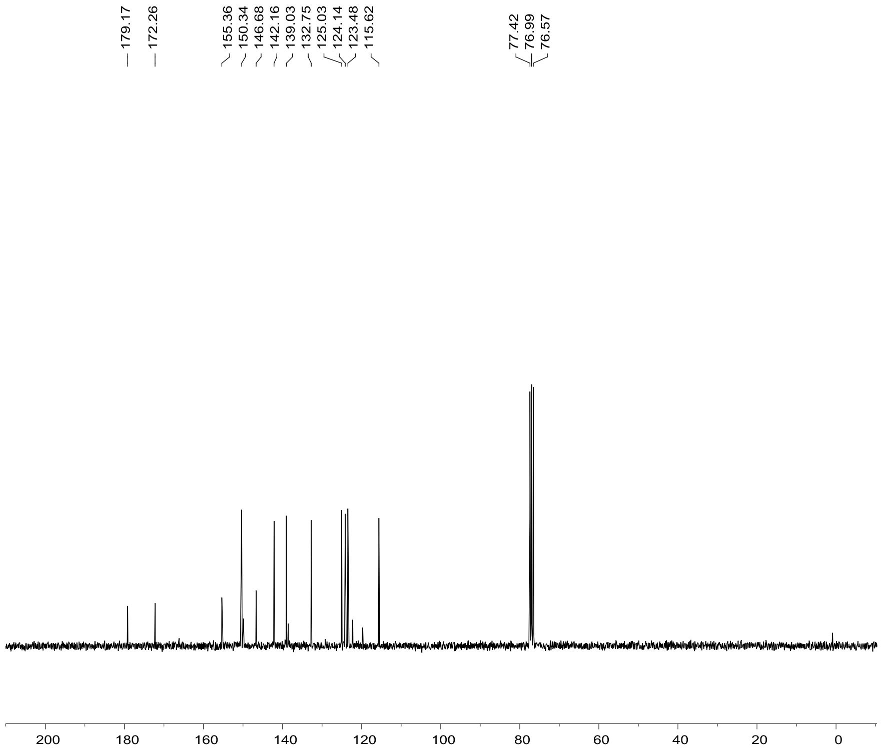

Parameter	Value
Title	zhanghao-13C.21.fid
Comment	13C NMR of zh-pydimer
Origin	UXNMR, Bruker Analytische Messtechnik GmbH
Owner	root
Site	
Spectrometer	dpx300
Author	
Solvent	CDCl3
Temperature	24.0
Pulse Sequence	zgdc30
Experiment	1D
Probe	5 mm PABBO BB-1H Z-GRD Z862701/ 0035
Number of Scans	223
Receiver Gain	4096
Relaxation Delay	1.5000
Pulse Width	12.0000
Acquisition Time	1.4451
Acquisition Date	2013-12-18T15:24:00
Modification Date	2013-12-18T15:35:36
Class	
Spectrometer Frequency	75.48
Spectral Width	22675.7
Lowest Frequency	-3801.7
Nucleus	13C
Acquired Size	32768
Spectral Size	65536

Figure S3. ^{1}H-NMR spectra of compound **4b**.

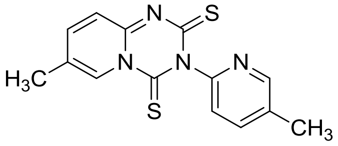

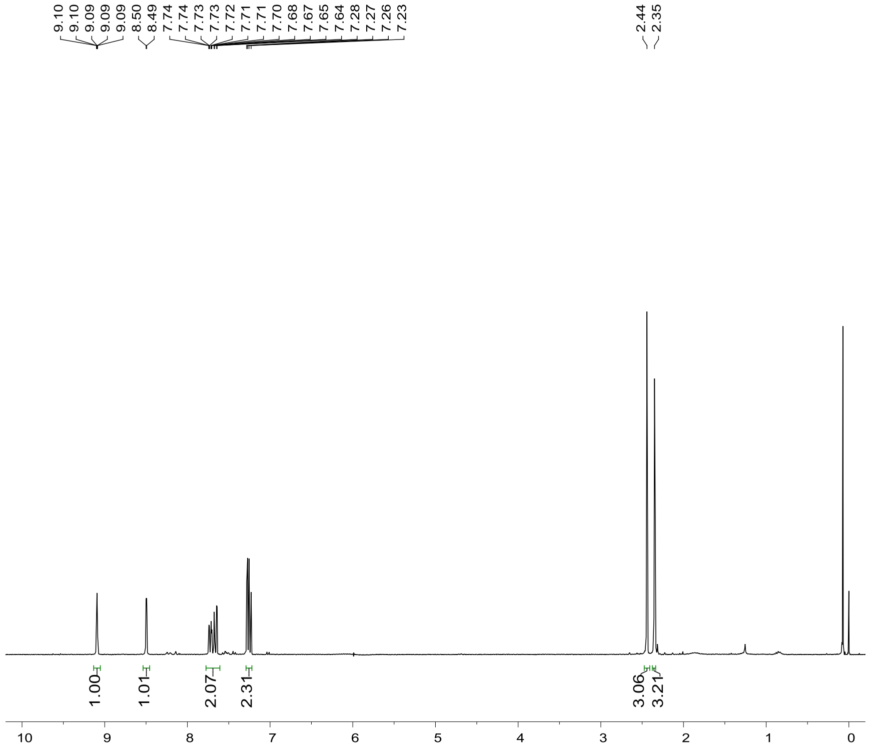

Parameter	Value
Title	zhanghao-H.43.fid
Comment	1H NMR of zh-mepyNCS
Origin	UXNMR, Bruker Analytische Messtechnik GmbH
Owner	root
Site	
Spectrometer	dpx300
Author	
Solvent	CDCl3
Temperature	24.0
Pulse Sequence	zg30
Experiment	1D
Probe	5 mm PABBO BB-1H Z-GRD Z862701/ 0035
Number of Scans	3
Receiver Gain	124
Relaxation Delay	2.0000
Pulse Width	10.7000
Acquisition Time	5.4658
Acquisition Date	2013-12-23T18:02:00
Modification Date	2013-12-23T18:02:33
Class	
Spectrometer Frequency	300.13
Spectral Width	5995.2
Lowest Frequency	-1199.8
Nucleus	1H
Acquired Size	32768
Spectral Size	65536

Figure S4. ^{13}C-NMR spectra of compound **4b**.

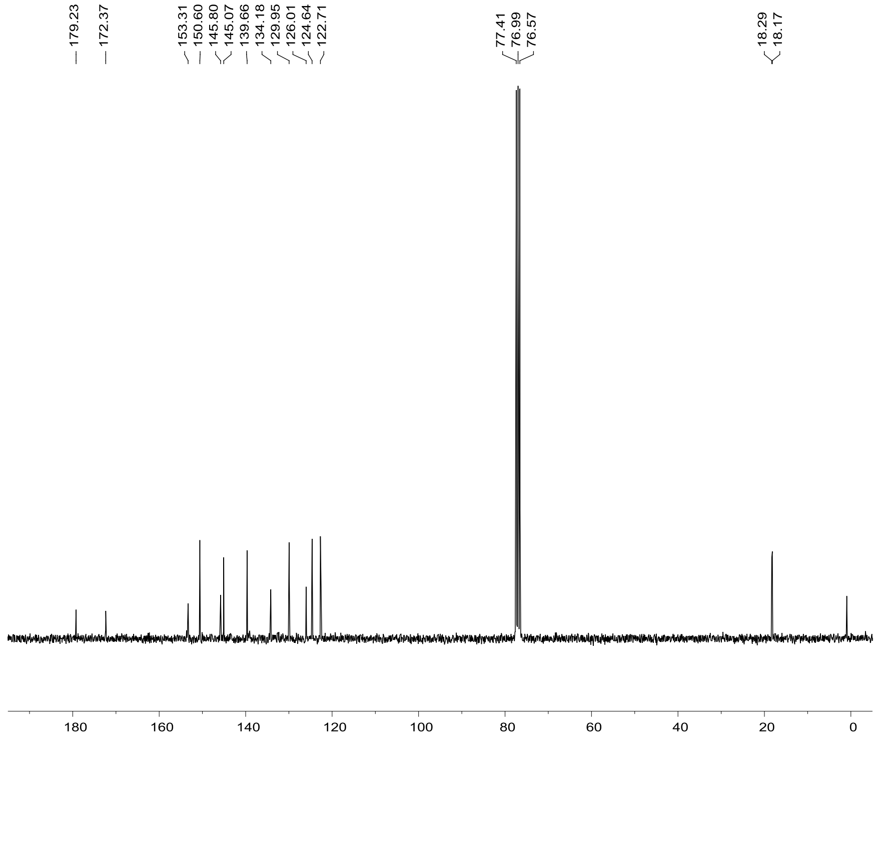

Parameter	Value
Title	zhanghao-13C.23.fid
Comment	13C NMR of zh-mepyNCS
Origin	UXNMR, Bruker Analytische Messtechnik GmbH
Owner	root
Site	
Spectrometer	dpx300
Author	
Solvent	CDCl3
Temperature	24.0
Pulse Sequence	zgdc30
Experiment	1D
Probe	5 mm PABBO BB-1H Z-GRD Z862701/ 0035
Number of Scans	880
Receiver Gain	9216
Relaxation Delay	1.5000
Pulse Width	12.0000
Acquisition Time	1.4451
Acquisition Date	2013-12-23T18:07:00
Modification Date	2013-12-23T18:51:20
Class	
Spectrometer Frequency	75.48
Spectral Width	22675.7
Lowest Frequency	-3795.4
Nucleus	13C
Acquired Size	32768
Spectral Size	65536

Figure S5. ^{1}H-NMR spectra of compound **4c**.

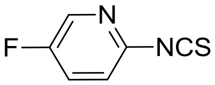

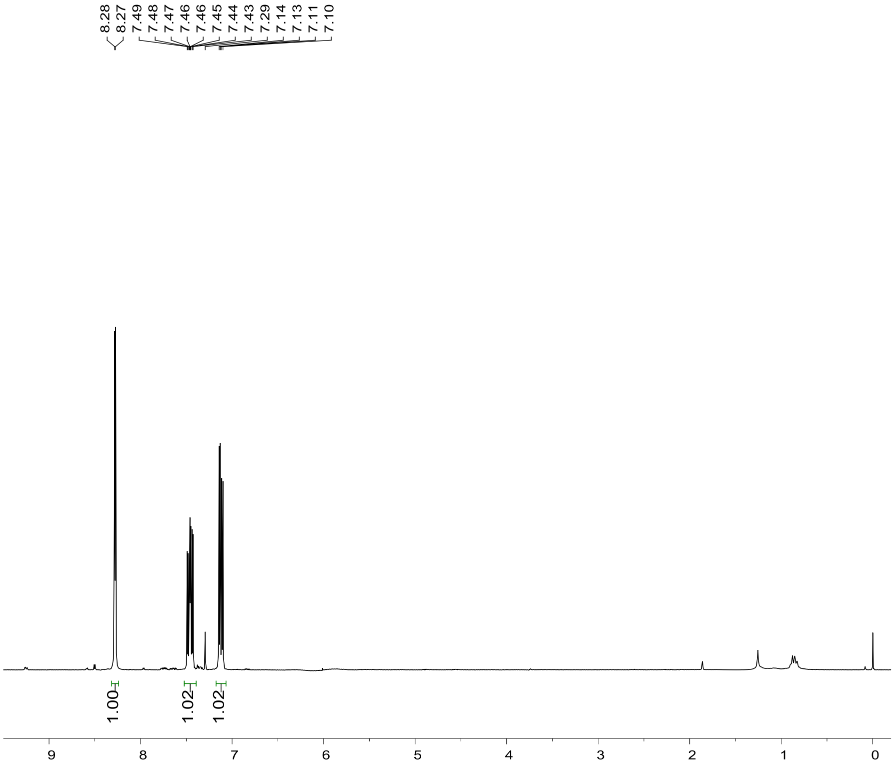

Parameter	Value
Title	zhanghao-H.27.fid
Comment	1H NMR of zh-FNCS
Origin	UXNMR, Bruker Analytische Messtechnik GmbH
Owner	root
Site	
Spectrometer	dpx300
Author	
Solvent	CDCl3
Temperature	24.0
Pulse Sequence	zg30
Experiment	1D
Probe	5 mm PABBO BB-1H Z-GRD Z862701/ 0035
Number of Scans	1
Receiver Gain	124
Relaxation Delay	2.0000
Pulse Width	10.7000
Acquisition Time	5.4658
Acquisition Date	2013-11-15T12:41:00
Modification Date	2013-11-15T12:41:48
Class	
Spectrometer Frequency	300.13
Spectral Width	5995.2
Lowest Frequency	-1193.1
Nucleus	1H
Acquired Size	32768
Spectral Size	65536

Figure S6. ^{13}C-NMR spectra of compound **4c**.

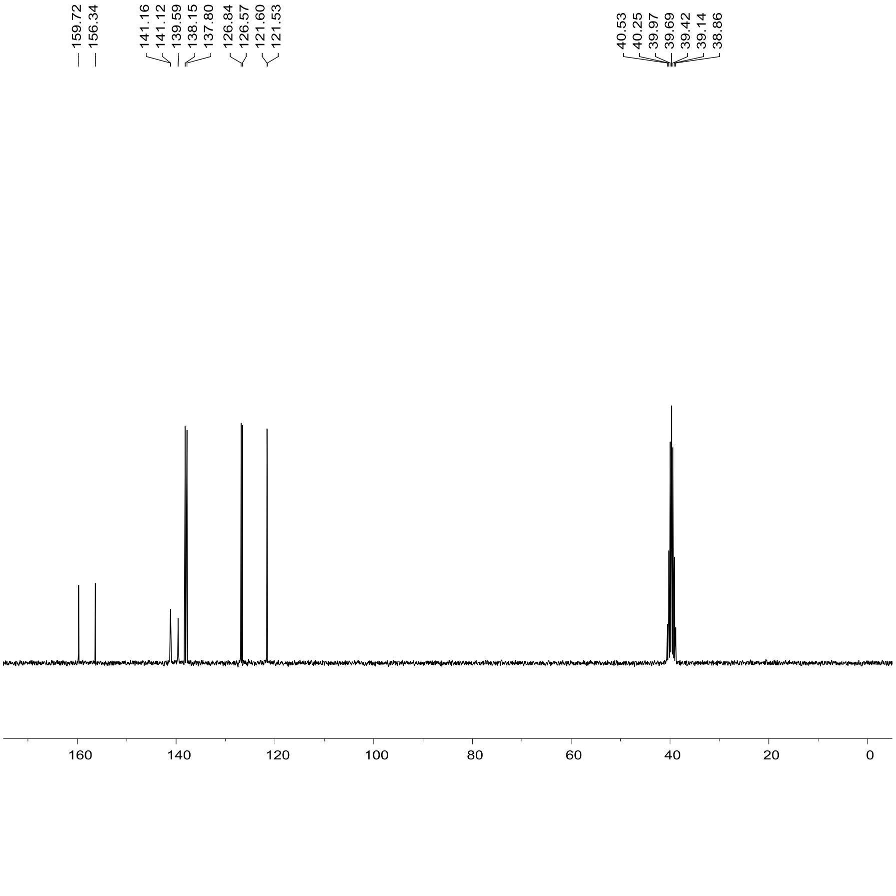

Parameter	Value
Title	zhanghao-13C.5.fid
Comment	13C NMR of zh-FPYNCS-DMSO
Origin	UXNMR, Bruker Analytische Messtechnik GmbH
Owner	root
Site	
Spectrometer	dpx300
Author	
Solvent	DMSO
Temperature	24.0
Pulse Sequence	zgdc30
Experiment	1D
Probe	5 mm PABBO BB-1H Z-GRD Z862701/ 0035
Number of Scans	410
Receiver Gain	4096
Relaxation Delay	1.5000
Pulse Width	12.0000
Acquisition Time	1.4451
Acquisition Date	2014-01-16T18:08:00
Modification Date	2014-01-16T18:27:51
Class	
Spectrometer Frequency	75.48
Spectral Width	22675.7
Lowest Frequency	-3818.1
Nucleus	13C
Acquired Size	32768
Spectral Size	65536

Figure S7. ^{1}H-NMR spectra of compound **4d**.

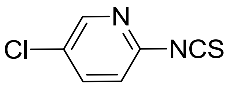

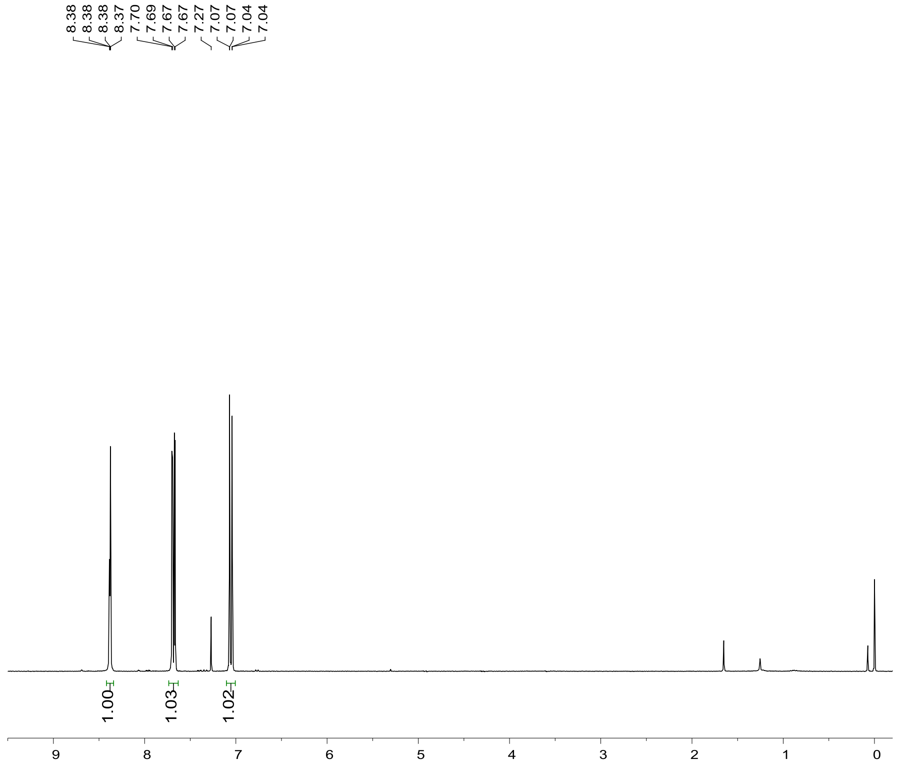

Parameter	Value
Title	zhanghao-H.29.fid
Comment	1H NMR of zh-clpyNCS
Origin	UXNMR, Bruker Analytische Messtechnik GmbH
Owner	root
Site	
Spectrometer	dpx300
Author	
Solvent	CDCl3
Temperature	24.0
Pulse Sequence	zg30
Experiment	1D
Probe	5 mm PABBO BB-1H Z-GRD Z862701/ 0035
Number of Scans	4
Receiver Gain	256
Relaxation Delay	2.0000
Pulse Width	10.7000
Acquisition Time	5.4658
Acquisition Date	2011-10-17T15:17:00
Modification Date	2011-10-17T15:17:26
Class	
Spectrometer Frequency	300.13
Spectral Width	5995.2
Lowest Frequency	-1200.0
Nucleus	1H
Acquired Size	32768
Spectral Size	65536

Figure S8. ^{13}C-NMR spectra of compound **4d**.

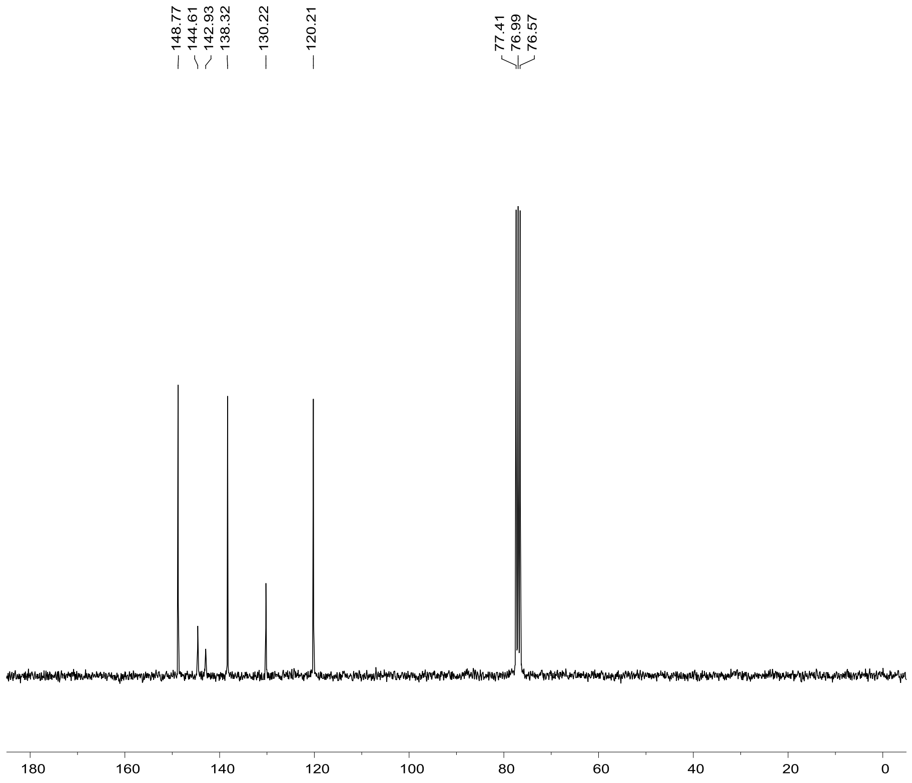

Parameter	Value
Title	zhanghao-13C.8.fid
Comment	13C NMR of zh-clNCS
Origin	UXNMR, Bruker Analytische Messtechnik GmbH
Owner	root
Site	
Spectrometer	dpx300
Author	
Solvent	CDCl3
Temperature	24.0
Pulse Sequence	zgdc30
Experiment	1D
Probe	5 mm PABBO BB-1H Z-GRD Z862701/ 0035
Number of Scans	503
Receiver Gain	5120
Relaxation Delay	1.5000
Pulse Width	12.0000
Acquisition Time	1.4451
Acquisition Date	2013-11-13T16:49:00
Modification Date	2013-11-13T17:14:52
Class	
Spectrometer Frequency	75.48
Spectral Width	22675.7
Lowest Frequency	-3794.4
Nucleus	13C
Acquired Size	32768
Spectral Size	65536

Figure S9. ^{1}H-NMR spectra of compound **4e**.

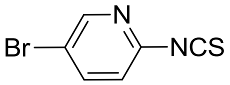

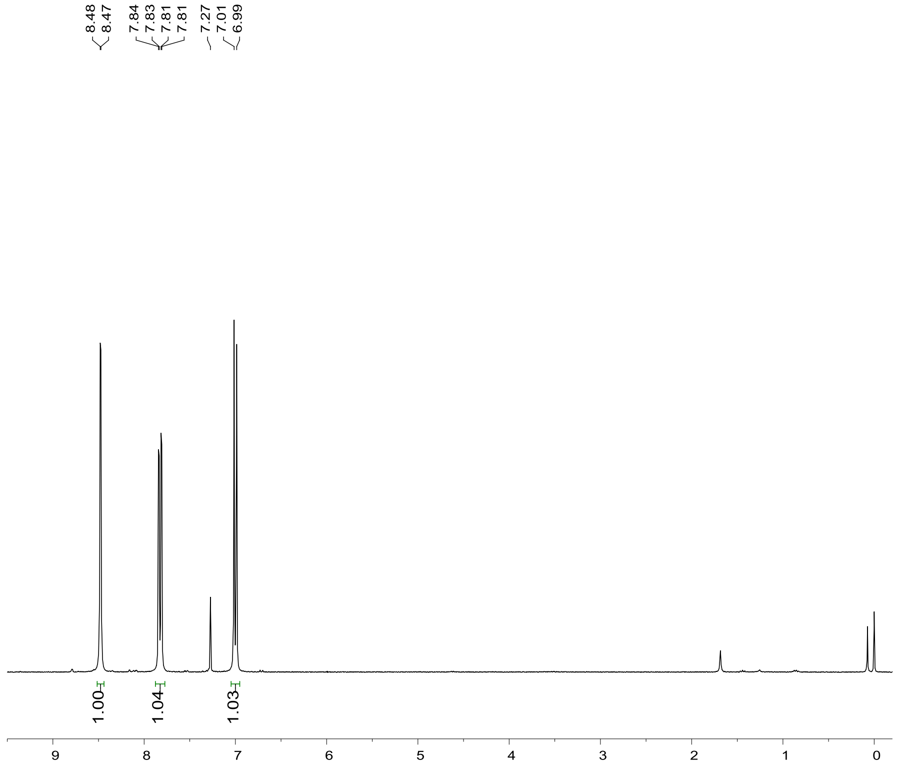

Parameter	Value
Title	zhanghao-H.32.fid
Comment	1H NMR of zh-BrNCS
Origin	UXNMR, Bruker Analytische Messtechnik GmbH
Owner	root
Site	
Spectrometer	dpx300
Author	
Solvent	CDCl3
Temperature	24.0
Pulse Sequence	zg30
Experiment	1D
Probe	5 mm PABBO BB-1H Z-GRD Z862701/ 0035
Number of Scans	6
Receiver Gain	256
Relaxation Delay	2.0000
Pulse Width	10.7000
Acquisition Time	5.4658
Acquisition Date	2013-11-25T14:46:00
Modification Date	2013-11-25T14:47:00
Class	
Spectrometer Frequency	300.13
Spectral Width	5995.2
Lowest Frequency	-1199.3
Nucleus	1H
Acquired Size	32768
Spectral Size	65536

Figure S10. ^{13}C-NMR spectra of compound **4e**.

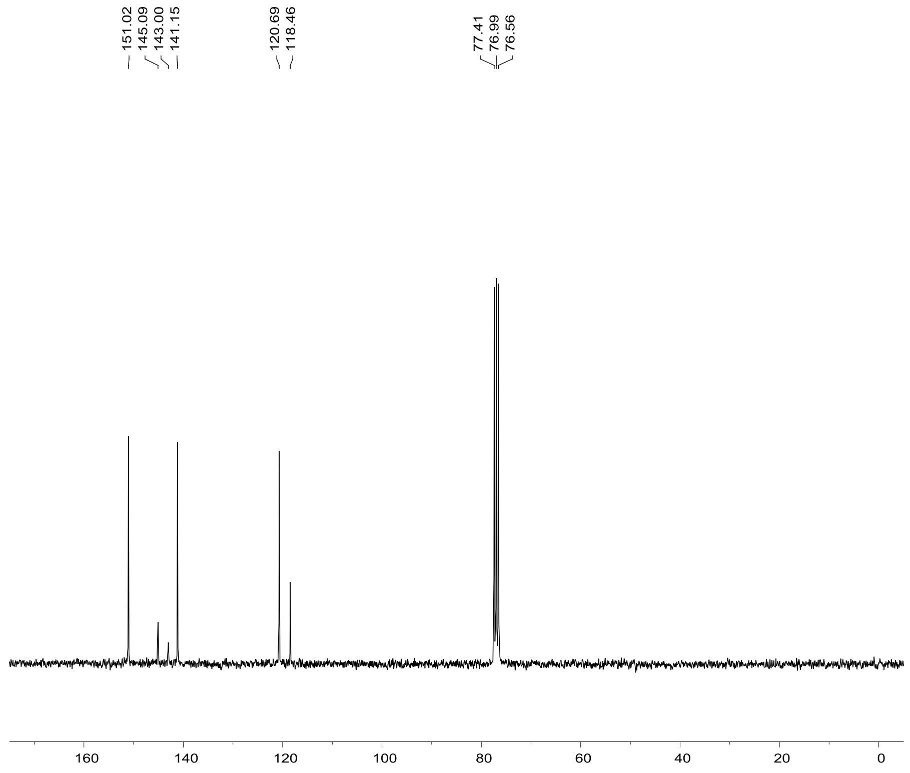

Parameter	Value
Title	zhanghao-13C.13.fid
Comment	13C NMR of zh-BrNCS
Origin	UXNMR, Bruker Analytische Messtechnik GmbH
Owner	root
Site	
Spectrometer	dpx300
Author	
Solvent	CDCl3
Temperature	24.0
Pulse Sequence	zgdc30
Experiment	1D
Probe	5 mm PABBO BB-1H Z-GRD Z862701/ 0035
Number of Scans	496
Receiver Gain	5120
Relaxation Delay	1.5000
Pulse Width	12.0000
Acquisition Time	1.4451
Acquisition Date	2013-11-25T21:54:00
Modification Date	2013-11-25T22:18:37
Class	
Spectrometer Frequency	75.48
Spectral Width	22675.7
Lowest Frequency	-3794.8
Nucleus	13C
Acquired Size	32768
Spectral Size	65536

Figure S11. ^{1}H-NMR spectra of compound **4f**.

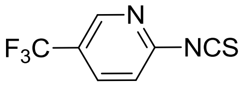

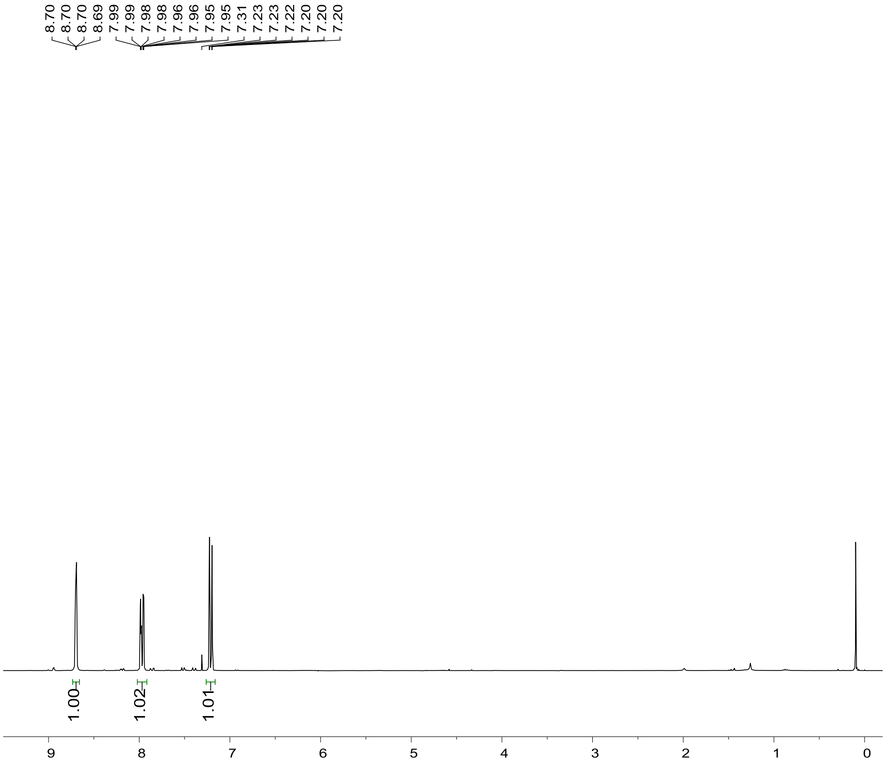

Parameter	Value
Title	zhanghao-H.44.fid
Comment	1H NMR of zh-CF3pyNCS
Origin	UXNMR, Bruker Analytische Messtechnik GmbH
Owner	root
Site	
Spectrometer	dpx300
Author	
Solvent	CDCl3
Temperature	24.0
Pulse Sequence	zg30
Experiment	1D
Probe	5 mm PABBO BB-1H Z-GRD Z862701/ 0035
Number of Scans	3
Receiver Gain	64
Relaxation Delay	2.0000
Pulse Width	10.7000
Acquisition Time	5.4658
Acquisition Date	2013-12-25T14:32:00
Modification Date	2013-12-25T14:33:12
Class	
Spectrometer Frequency	300.13
Spectral Width	5995.2
Lowest Frequency	-1188.3
Nucleus	1H
Acquired Size	32768
Spectral Size	65536

Figure S12. ^{13}C-NMR spectra of compound **4f**.

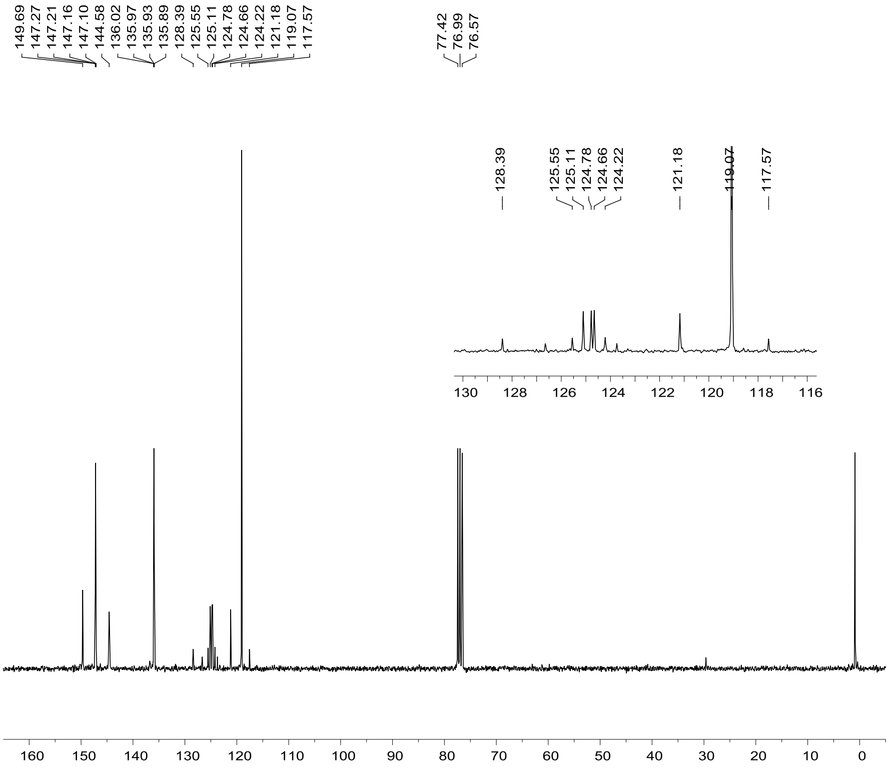

Parameter	Value
Title	zhanghao-13C. 24.fid
Comment	13C NMR of zh-CF3pyNCS
Origin	UXNMR, Bruker Analytische Messtechnik GmbH
Owner	root
Site	
Spectrometer	dpx300
Author	
Solvent	CDCl3
Temperature	24.0
Pulse Sequence	zgdc30
Experiment	1D
Probe	5 mm PABBO BB-1H Z-GRD Z862701/ 0035
Number of Scans	538
Receiver Gain	5120
Relaxation Delay	1.5000
Pulse Width	12.0000
Acquisition Time	1.4451
Acquisition Date	2013-12-25T20 :29:00
Modification Date	2013-12-25T20 :39:33
Class	
Spectrometer Frequency	75.48
Spectral Width	22675.7
Lowest Frequency	-3795.4
Nucleus	13C
Acquired Size	32768
Spectral Size	65536

Figure S13. ^{1}H-NMR spectra of compound **4g**.

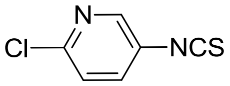

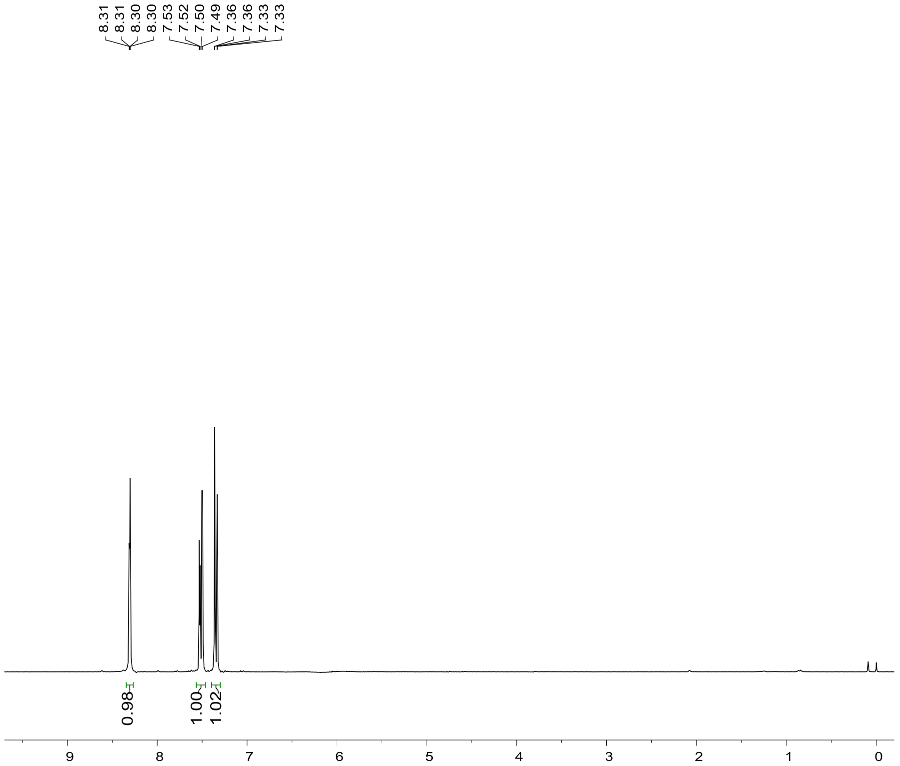

Parameter	Value
Title	zhanghao-H.36.fid
Comment	1H NMR of zh-3cl2NNCS
Origin	UXNMR, Bruker Analytische Messtechnik GmbH
Owner	root
Site	
Spectrometer	dpx300
Author	
Solvent	CDCl3
Temperature	24.0
Pulse Sequence	zg30
Experiment	1D
Probe	5 mm PABBO BB-1H Z-GRD Z862701/ 0035
Number of Scans	1
Receiver Gain	64
Relaxation Delay	2.0000
Pulse Width	10.7000
Acquisition Time	5.4658
Acquisition Date	2013-12-03T12:03:00
Modification Date	2013-12-03T12:04:04
Class	
Spectrometer Frequency	300.13
Spectral Width	5995.2
Lowest Frequency	-1179.9
Nucleus	1H
Acquired Size	32768
Spectral Size	65536

Figure S14. ^{13}C-NMR spectra of compound **4g**.

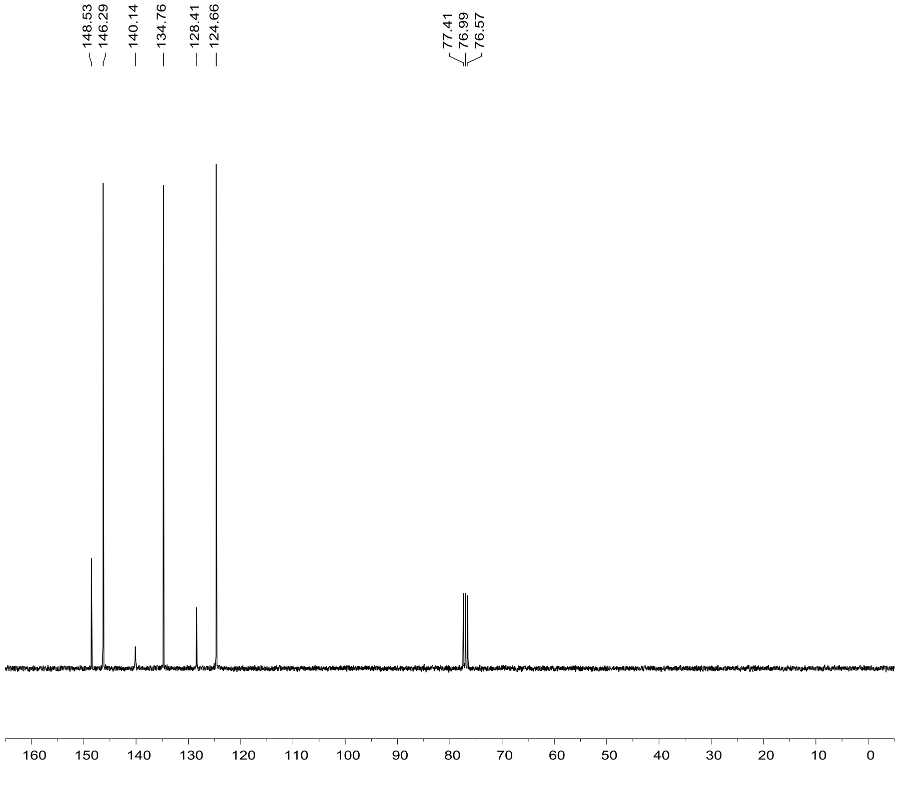

Parameter	Value
Title	zhanghao-13C.19.fid
Comment	13C NMR of zh-3Cl2NNCS
Origin	UXNMR, Bruker Analytische Messtechnik GmbH
Owner	root
Site	
Spectrometer	dpx300
Author	
Solvent	CDCl3
Temperature	25.0
Pulse Sequence	zgdc30
Experiment	1D
Probe	5 mm PABBO BB-1H Z-GRD Z862701/ 0035
Number of Scans	128
Receiver Gain	2048
Relaxation Delay	1.5000
Pulse Width	12.0000
Acquisition Time	1.4451
Acquisition Date	2013-12-03T21:27:00
Modification Date	2013-12-03T21:33:38
Class	
Spectrometer Frequency	75.48
Spectral Width	22675.7
Lowest Frequency	-3810.0
Nucleus	13C
Acquired Size	32768
Spectral Size	65536

Figure S15. ^{1}H-NMR spectra of compound **4h**.

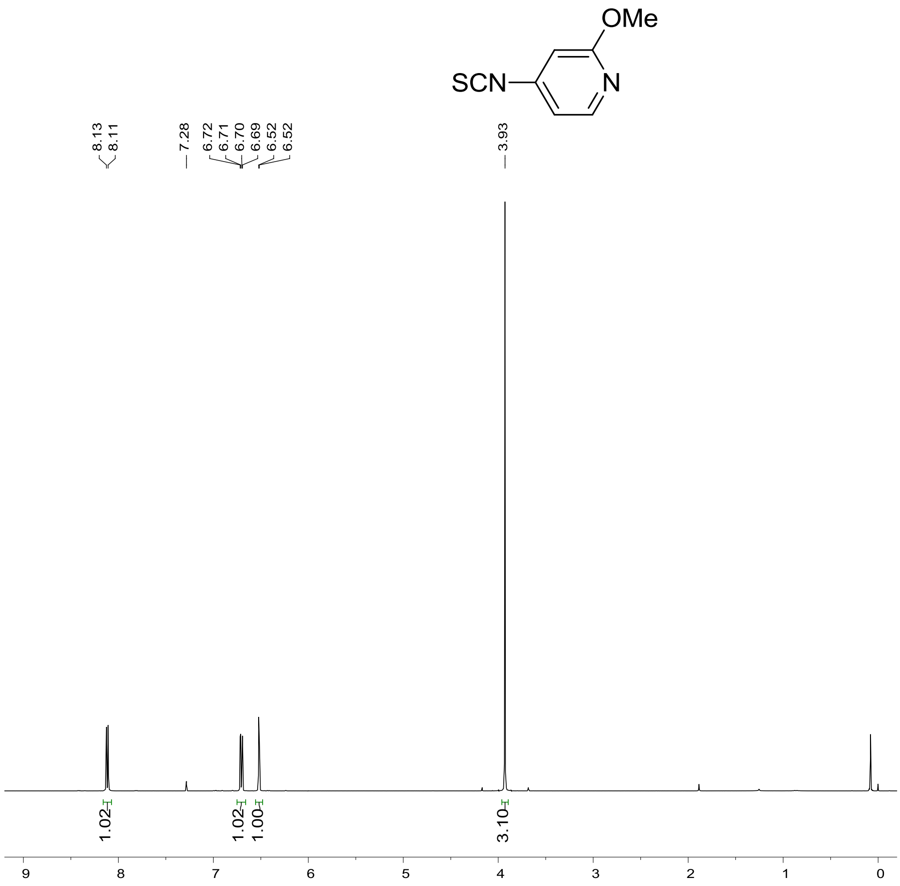

Parameter	Value
Title	zhanghao-H.26.fid
Comment	1H NMR of zh-OMeNCS
Origin	UXNMR, Bruker Analytische Messtechnik GmbH
Owner	root
Site	
Spectrometer	dpx300
Author	
Solvent	CDCl3
Temperature	24.0
Pulse Sequence	zg30
Experiment	1D
Probe	5 mm PABBO BB-1H Z-GRD Z862701/ 0035
Number of Scans	8
Receiver Gain	64
Relaxation Delay	2.0000
Pulse Width	10.7000
Acquisition Time	5.4658
Acquisition Date	2013-11-14T19:35:00
Modification Date	2013-11-14T19:35:28
Class	
Spectrometer Frequency	300.13
Spectral Width	5995.2
Lowest Frequency	-1196.0
Nucleus	1H
Acquired Size	32768
Spectral Size	65536

Figure S16. ^{13}C-NMR spectra of compound **4h**.

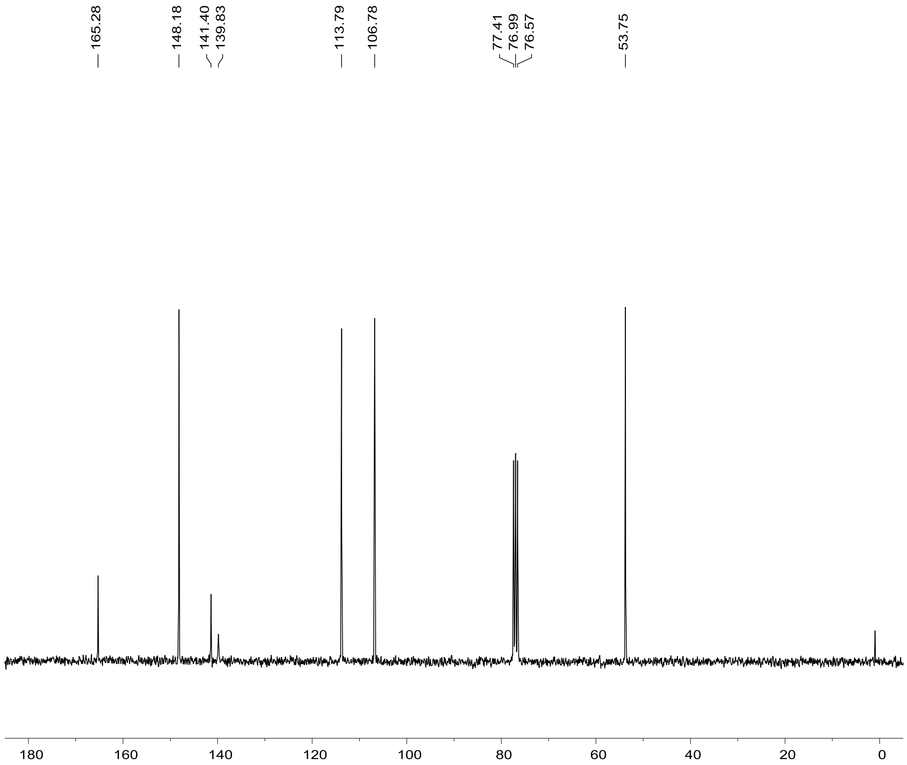

Parameter	Value
Title	zhanghao-13C.9.fid
Comment	13C NMR of zh-OMeNCS
Origin	UXNMR, Bruker Analytische Messtechnik GmbH
Owner	root
Site	
Spectrometer	dpx300
Author	
Solvent	CDCl3
Temperature	24.0
Pulse Sequence	zgdc30
Experiment	1D
Probe	5 mm PABBO BB-1H Z-GRD Z862701/ 0035
Number of Scans	256
Receiver Gain	7168
Relaxation Delay	1.5000
Pulse Width	12.0000
Acquisition Time	1.4451
Acquisition Date	2013-11-14T19:39:00
Modification Date	2013-11-14T19:51:51
Class	
Spectrometer Frequency	75.48
Spectral Width	22675.7
Lowest Frequency	-3795.1
Nucleus	13C
Acquired Size	32768
Spectral Size	65536

Figure S17. ^{1}H-NMR spectra of compound 4i.

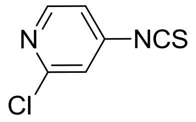

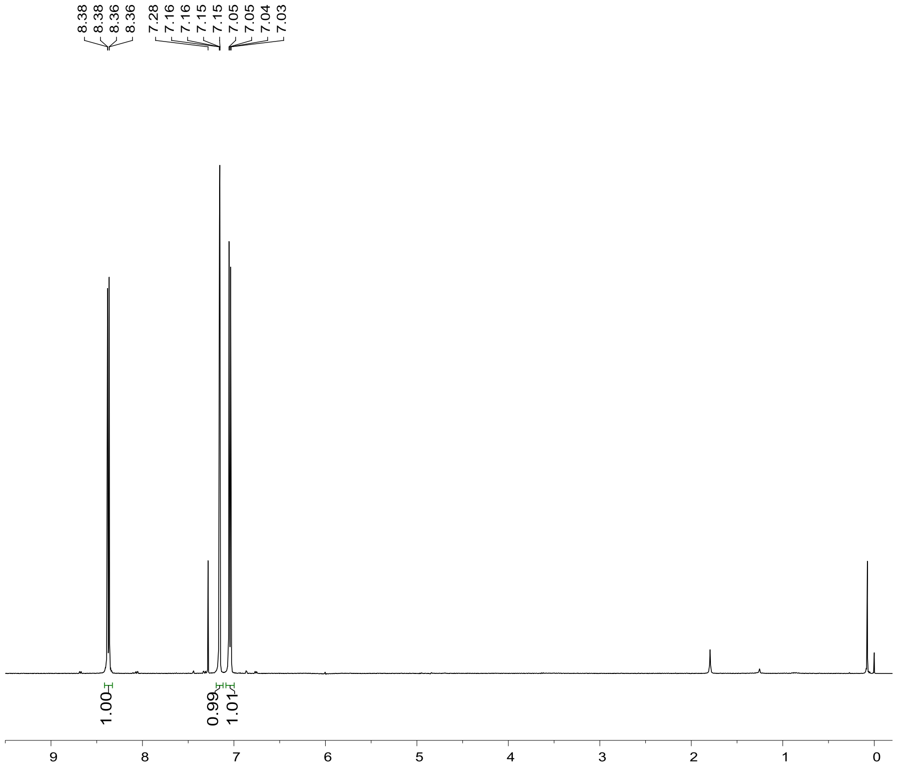

Parameter	Value
Title	zhanghao-H.3.fid
Comment	1H NMR of 2cl4NCS
Origin	UXNMR, Bruker Analytische Messtechnik GmbH
Owner	root
Site	
Spectrometer	dpx300
Author	
Solvent	CDCl3
Temperature	24.0
Pulse Sequence	zg30
Experiment	1D
Probe	5 mm PABBO BB-1H Z-GRD Z862701/ 0035
Number of Scans	5
Receiver Gain	124
Relaxation Delay	2.0000
Pulse Width	10.7000
Acquisition Time	5.4658
Acquisition Date	2014-03-17T10:04:00
Modification Date	2014-03-17T10:05:26
Class	
Spectrometer Frequency	300.13
Spectral Width	5995.2
Lowest Frequency	-1196.2
Nucleus	1H
Acquired Size	32768
Spectral Size	65536

Figure S18. ^{13}C-NMR spectra of compound 4i.

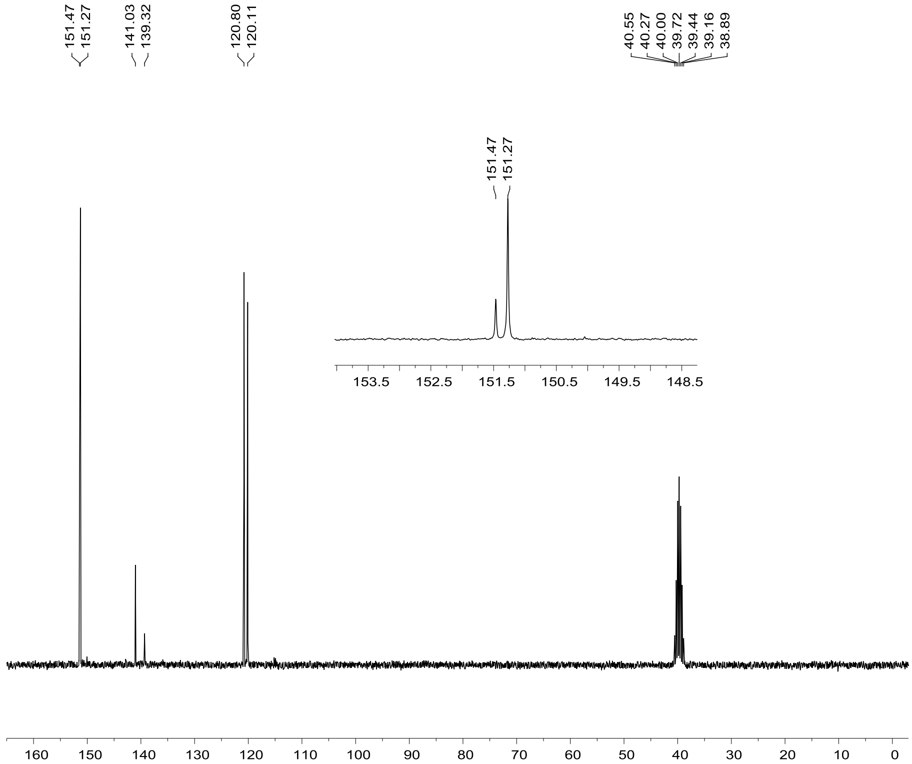

Parameter	Value
Title	zhanghao-13C.2.fid
Comment	13C NMR of zh-2cl4NCS
Origin	UXNMR, Bruker Analytische Messtechnik GmbH
Owner	root
Site	
Spectrometer	dpx300
Author	
Solvent	DMSO
Temperature	24.0
Pulse Sequence	zgdc30
Experiment	1D
Probe	5 mm PABBO BB-1H Z-GRD Z862701/ 0035
Number of Scans	128
Receiver Gain	6144
Relaxation Delay	1.5000
Pulse Width	12.0000
Acquisition Time	1.4451
Acquisition Date	2014-08-19T14:46:00
Modification Date	2014-08-19T14:52:55
Class	
Spectrometer Frequency	75.48
Spectral Width	22675.7
Lowest Frequency	-3818.8
Nucleus	13C
Acquired Size	32768
Spectral Size	65536

Figure S19. ^{1}H-NMR spectra of compound **4j**.

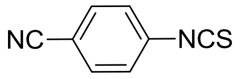

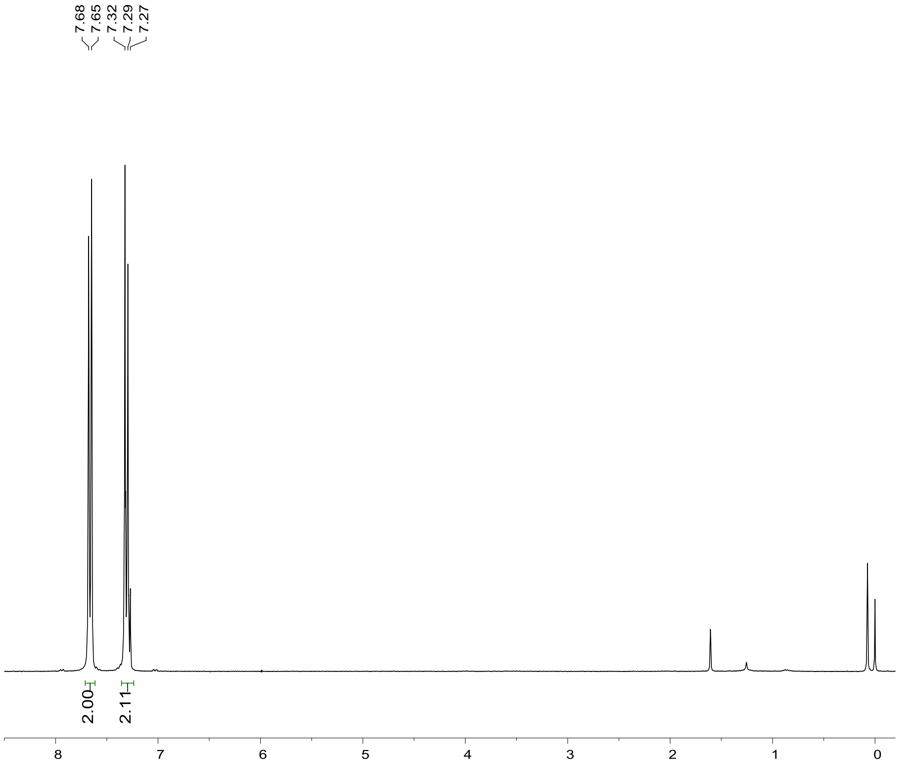

Parameter	Value
Title	zhanghao-H.21.fid
Comment	1H NMR of zh-CNNCS
Origin	UXNMR, Bruker Analytische Messtechnik GmbH
Owner	root
Site	
Spectrometer	dpx300
Author	
Solvent	CDC13
Temperature	24.0
Pulse Sequence	zg30
Experiment	1D
Probe	5 mm PABBO BB-1H Z-GRD Z862701/ 0035
Number of Scans	10
Receiver Gain	156
Relaxation Delay	2.0000
Pulse Width	10.7000
Acquisition Time	5.4658
Acquisition Date	2013-11-07T14:04:00
Modification Date	2013-11-07T14:05:22
Class	
Spectrometer Frequency	300.13
Spectral Width	5995.2
Lowest Frequency	-1200.0
Nucleus	1H
Acquired Size	32768
Spectral Size	65536

Figure S20. ^{13}C-NMR spectra of compound **4j**.

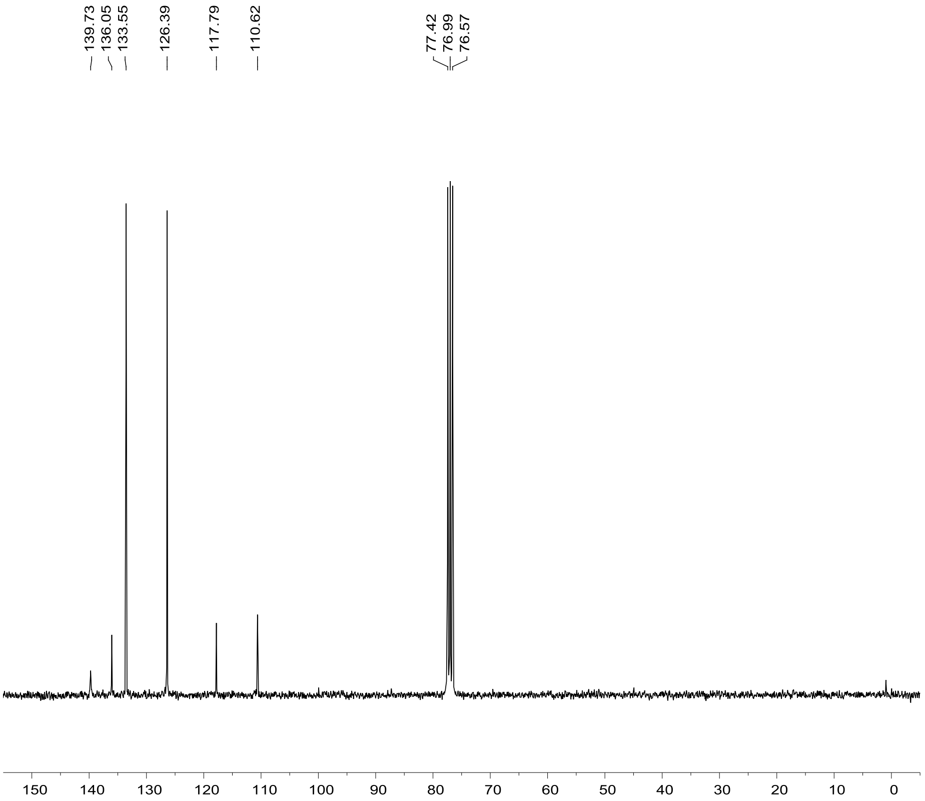

Parameter	Value
Title	zhanghao-13C.4.fid
Comment	13C NMR of zh-CN NCS
Origin	UXNMR, Bruker Analytische Messtechnik GmbH
Owner	root
Site	
Spectrometer	dpx300
Author	
Solvent	CDCl3
Temperature	24.0
Pulse Sequence	zgdc30
Experiment	1D
Probe	5 mm PABBO BB-1H Z-GRD Z862701/ 0035
Number of Scans	1008
Receiver Gain	8192
Relaxation Delay	1.5000
Pulse Width	12.0000
Acquisition Time	1.4451
Acquisition Date	2013-11-07T19:28:00
Modification Date	2013-11-07T20:15:22
Class	
Spectrometer Frequency	75.48
Spectral Width	22675.7
Lowest Frequency	-3795.1
Nucleus	13C
Acquired Size	32768
Spectral Size	65536

Figure S21. ^{1}H-NMR spectra of compound **4k**.

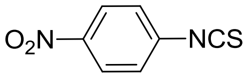

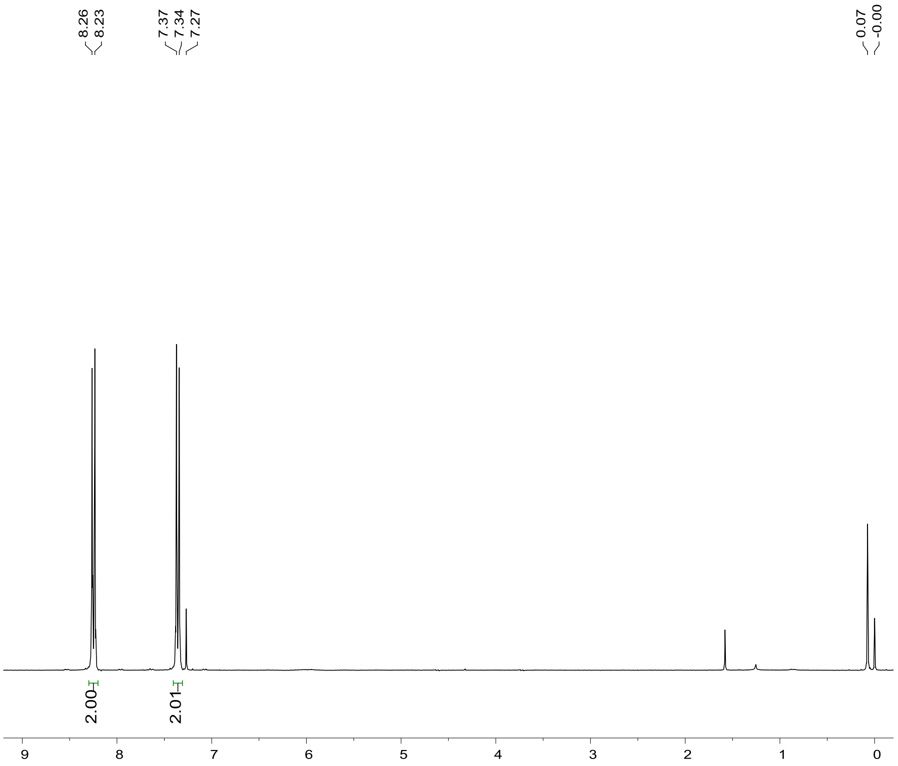

Parameter	Value
Title	zhanghao-H.22.fid
Comment	1H NMR of zh-NO2NCS
Origin	UXNMR, Bruker Analytische Messtechnik GmbH
Owner	root
Site	
Spectrometer	dpx300
Author	
Solvent	CDCl3
Temperature	24.0
Pulse Sequence	zg30
Experiment	1D
Probe	5 mm PABBO BB-1H Z-GRD Z862701/ 0035
Number of Scans	3
Receiver Gain	156
Relaxation Delay	2.0000
Pulse Width	10.7000
Acquisition Time	5.4658
Acquisition Date	2013-11-08T13:34:00
Modification Date	2013-11-08T13:34:38
Class	
Spectrometer Frequency	300.13
Spectral Width	5995.2
Lowest Frequency	-1200.8
Nucleus	1H
Acquired Size	32768
Spectral Size	65536

Figure S22. ^{13}C-NMR spectra of compound **4k**.

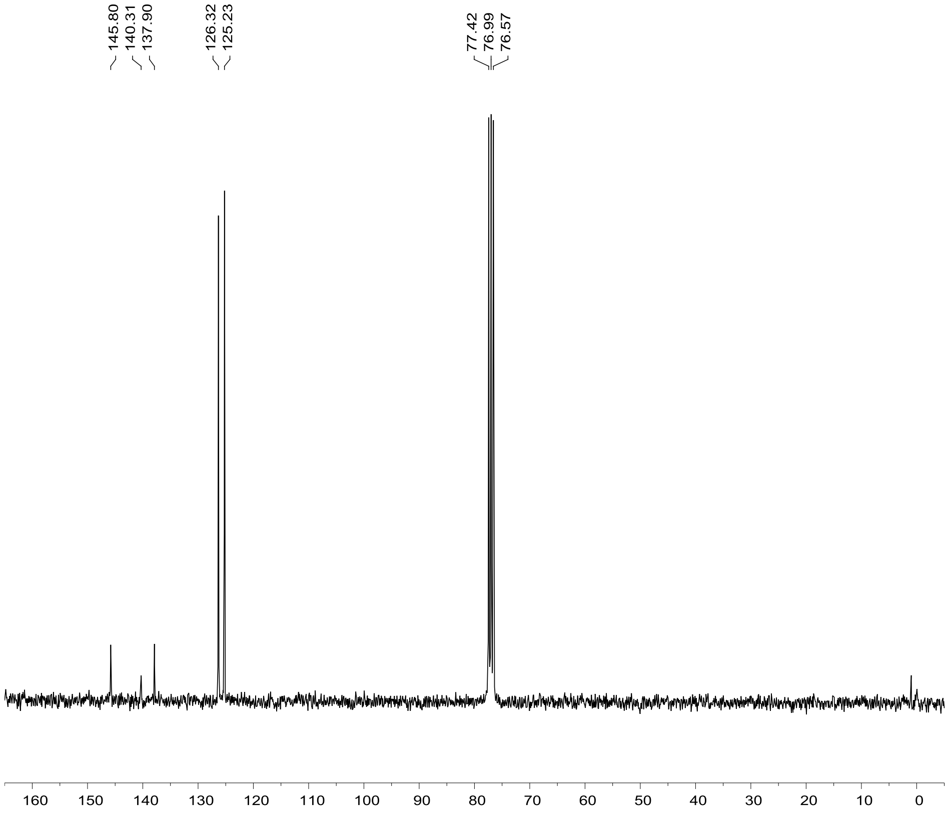

Parameter	Value
Title	zhanghao-13C.6.fid
Comment	13C NMR of zh-NO2NCS
Origin	UXNMR, Bruker Analytische Messtechnik GmbH
Owner	root
Site	
Spectrometer	dpx300
Author	
Solvent	CDCl3
Temperature	24.0
Pulse Sequence	zgdc30
Experiment	1D
Probe	5 mm PABBO BB-1H Z-GRD Z862701/ 0035
Number of Scans	372
Receiver Gain	7168
Relaxation Delay	1.5000
Pulse Width	12.0000
Acquisition Time	1.4451
Acquisition Date	2013-11-09T18:55:00
Modification Date	2013-11-09T19:03:26
Class	
Spectrometer Frequency	75.48
Spectral Width	22675.7
Lowest Frequency	-3793.4
Nucleus	13C
Acquired Size	32768
Spectral Size	65536

Figure S23. ^{1}H-NMR spectra of compound **4l**.

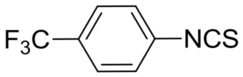

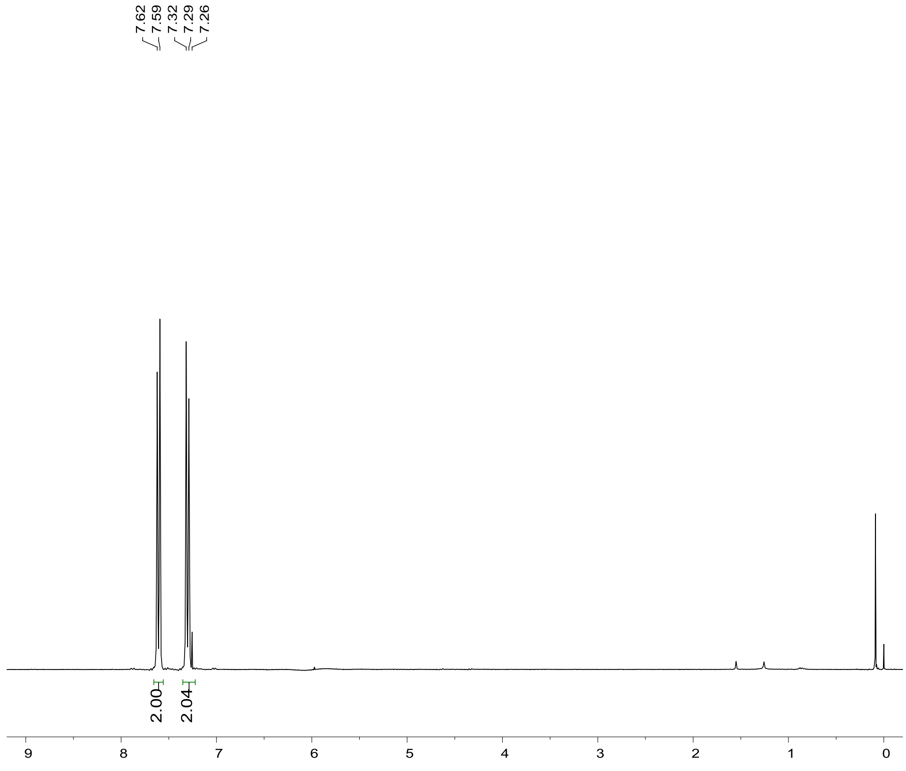

Parameter	Value
Title	zhanghao-H.42.fid
Comment	1H NMR of zh-CF3PhNCS
Origin	UXNMR, Bruker Analytische Messtechnik GmbH
Owner	root
Site	
Spectrometer	dpx300
Author	
Solvent	CDCl3
Temperature	24.0
Pulse Sequence	zg30
Experiment	1D
Probe	5 mm PABBO BB-1H Z-GRD Z862701/ 0035
Number of Scans	1
Receiver Gain	64
Relaxation Delay	2.0000
Pulse Width	10.7000
Acquisition Time	5.4658
Acquisition Date	2013-12-19T16:04:00
Modification Date	2013-12-19T16:04:20
Class	
Spectrometer Frequency	300.13
Spectral Width	5995.2
Lowest Frequency	-1204.6
Nucleus	1H
Acquired Size	32768
Spectral Size	65536

Figure S24. ^{13}C-NMR spectra of compound **4l**.

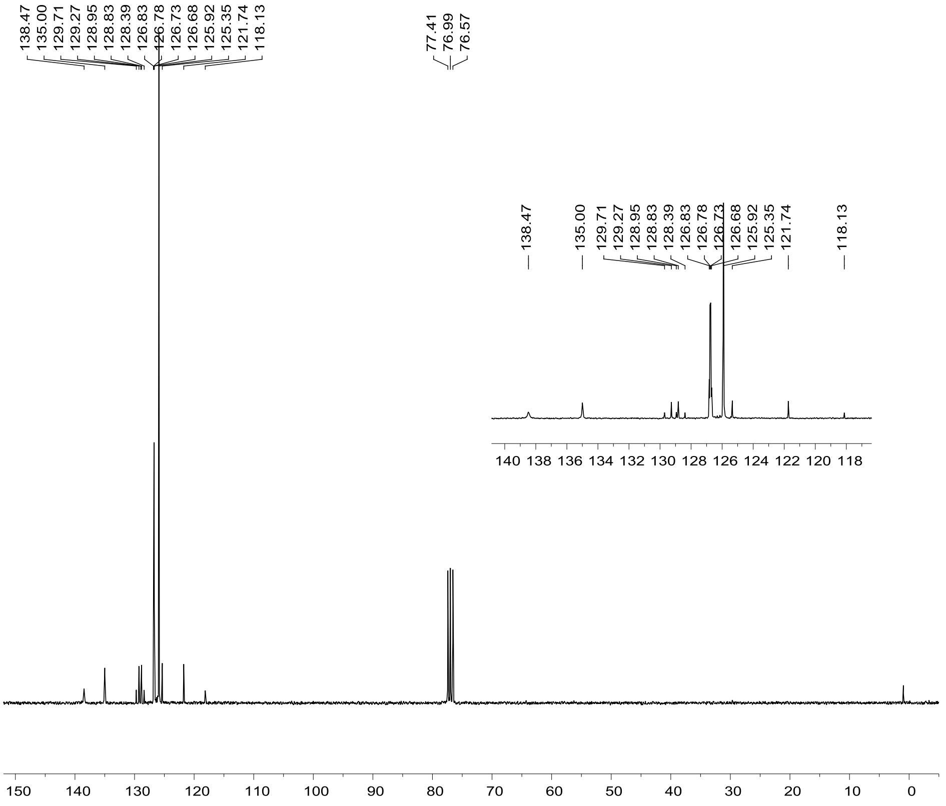

Parameter	Value
Title	zhanghao-13C.22.fid
Comment	13C NMR zh-CF3PhNCS
Origin	UXNMR, Bruker Analytische Messtechnik GmbH
Owner	root
Site	
Spectrometer	dpx300
Author	
Solvent	CDCl3
Temperature	24.0
Pulse Sequence	zgdc30
Experiment	1D
Probe	5 mm PABBO BB-1H Z-GRD Z862701/ 0035
Number of Scans	699
Receiver Gain	8192
Relaxation Delay	1.5000
Pulse Width	12.0000
Acquisition Time	1.4451
Acquisition Date	2013-12-21T10:14:00
Modification Date	2013-12-21T10:43:36
Class	
Spectrometer Frequency	75.48
Spectral Width	22675.7
Lowest Frequency	-3793.4
Nucleus	13C
Acquired Size	32768
Spectral Size	65536

Figure S25. ^{1}H-NMR spectra of compound **4m**.

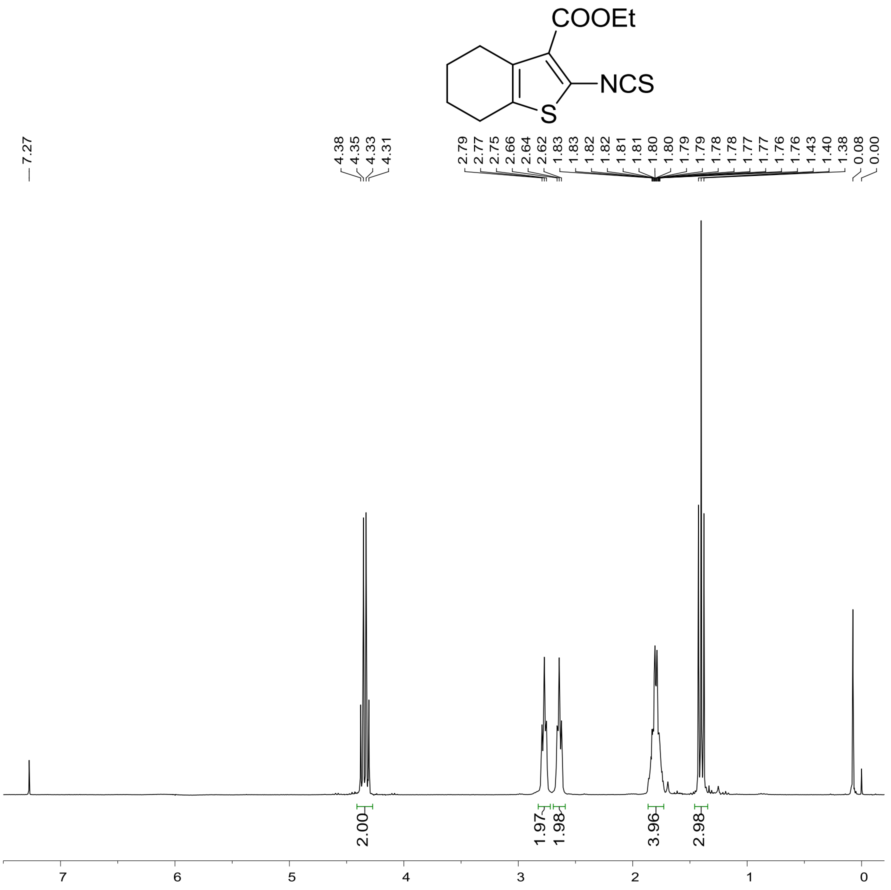

Parameter	Value
Title	zhanghao-H.1.fid
Comment	1H NMR of zh-saifenNCS
Origin	UXNMR, Bruker Analytische Messtechnik GmbH
Owner	root
Site	
Spectrometer	dpx300
Author	
Solvent	CDCl3
Temperature	24.0
Pulse Sequence	zg30
Experiment	1D
Probe	5 mm PABBO BB-1H Z-GRD Z862701/ 0035
Number of Scans	3
Receiver Gain	64
Relaxation Delay	2.0000
Pulse Width	10.7000
Acquisition Time	5.4658
Acquisition Date	2014-01-06T10:52:00
Modification Date	2014-01-06T10:53:07
Class	
Spectrometer Frequency	300.13
Spectral Width	5995.2
Lowest Frequency	-1198.0
Nucleus	1H
Acquired Size	32768
Spectral Size	65536

Figure S26. ^{13}C-NMR spectra of compound **4m**.

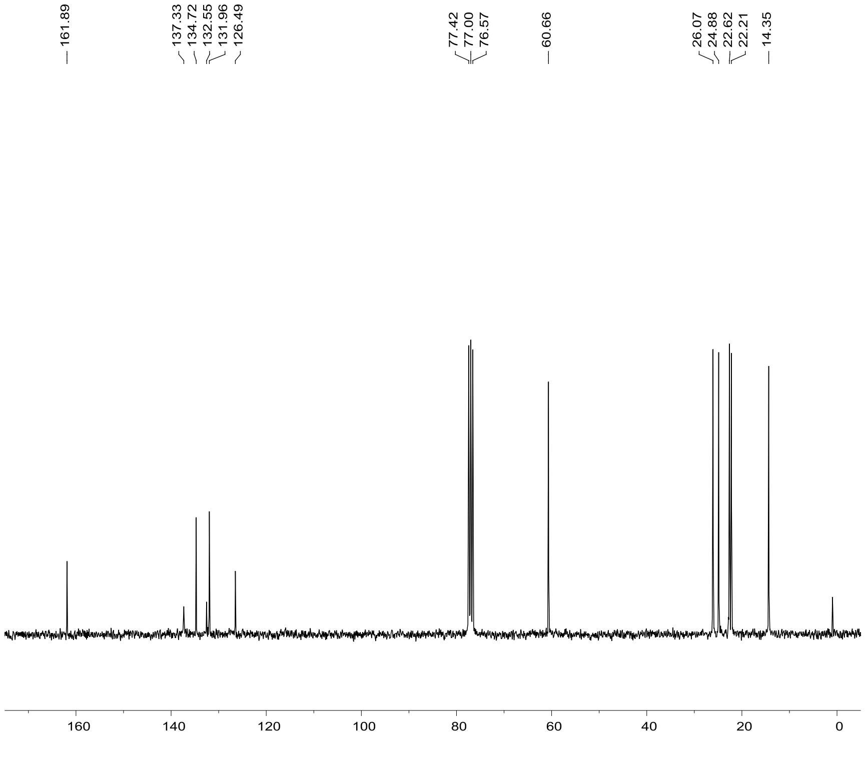

Parameter	Value
Title	zhanghao-13C.2.fid
Comment	13C NMR of saifenNCS
Origin	UXNMR, Bruker Analytische Messtechnik GmbH
Owner	root
Site	
Spectrometer	dpx300
Author	
Solvent	CDCl3
Temperature	24.0
Pulse Sequence	zgdc30
Experiment	1D
Probe	5 mm PABBO BB-1H Z-GRD Z862701/ 0035
Number of Scans	286
Receiver Gain	4096
Relaxation Delay	1.5000
Pulse Width	12.0000
Acquisition Time	1.4451
Acquisition Date	2014-01-07T18:28:00
Modification Date	2014-01-07T18:40:02
Class	
Spectrometer Frequency	75.48
Spectral Width	22675.7
Lowest Frequency	-3796.1
Nucleus	13C
Acquired Size	32768
Spectral Size	65536

Figure S27. ^{1}H-NMR spectra of compound **4n**.

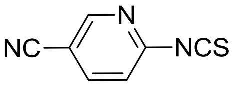

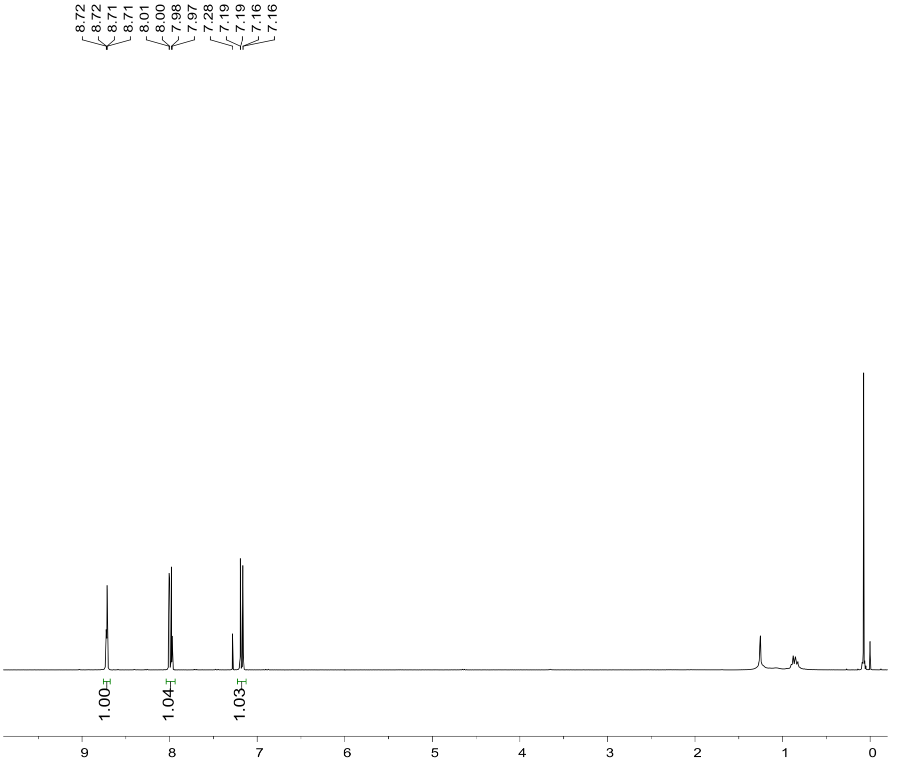

Parameter	Value
Title	zhanghao-H.34.fid
Comment	1H NMR of zh-CNPYNCS
Origin	UXNMR, Bruker Analytische Messtechnik GmbH
Owner	root
Site	
Spectrometer	dpx300
Author	
Solvent	CDCl3
Temperature	24.2
Pulse Sequence	zg30
Experiment	1D
Probe	5 mm PABBO BB-1H Z-GRD Z862701/ 0035
Number of Scans	10
Receiver Gain	128
Relaxation Delay	2.0000
Pulse Width	10.7000
Acquisition Time	5.4658
Acquisition Date	2013-11-26T16:09:00
Modification Date	2013-11-26T16:10:33
Class	
Spectrometer Frequency	300.13
Spectral Width	5995.2
Lowest Frequency	-1197.1
Nucleus	1H
Acquired Size	32768
Spectral Size	65536

Figure S28. ^{13}C-NMR spectra of compound **4n**.

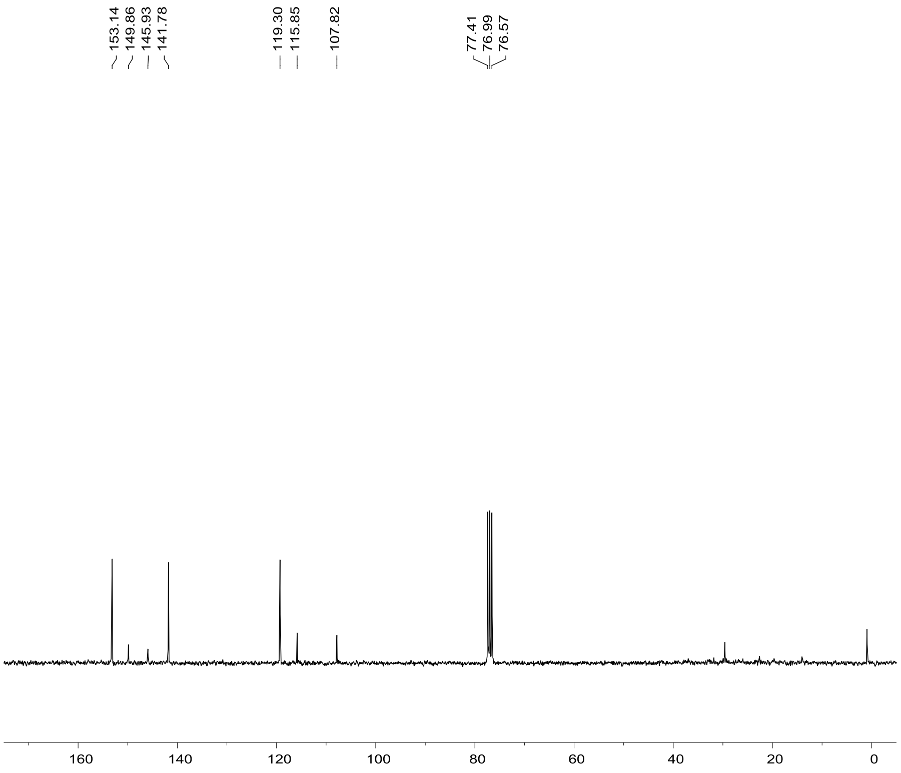

Parameter	Value
Title	zhanghao-13C.15.fid
Comment	13C NMR of zh-CNPYNCS
Origin	UXNMR, Bruker Analytische Messtechnik GmbH
Owner	root
Site	
Spectrometer	dpx300
Author	
Solvent	CDCl3
Temperature	25.2
Pulse Sequence	zgdc30
Experiment	1D
Probe	5 mm PABBO BB-1H Z-GRD Z862701/ 0035
Number of Scans	322
Receiver Gain	4096
Relaxation Delay	1.5000
Pulse Width	12.0000
Acquisition Time	1.4451
Acquisition Date	2013-11-26T19:00:00
Modification Date	2013-11-26T19:14:55
Class	
Spectrometer Frequency	75.48
Spectral Width	22675.7
Lowest Frequency	-3797.5
Nucleus	13C
Acquired Size	32768
Spectral Size	65536

Figure S29. ^{1}H-NMR spectra of compound **4o**.

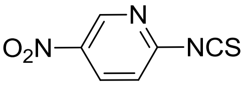

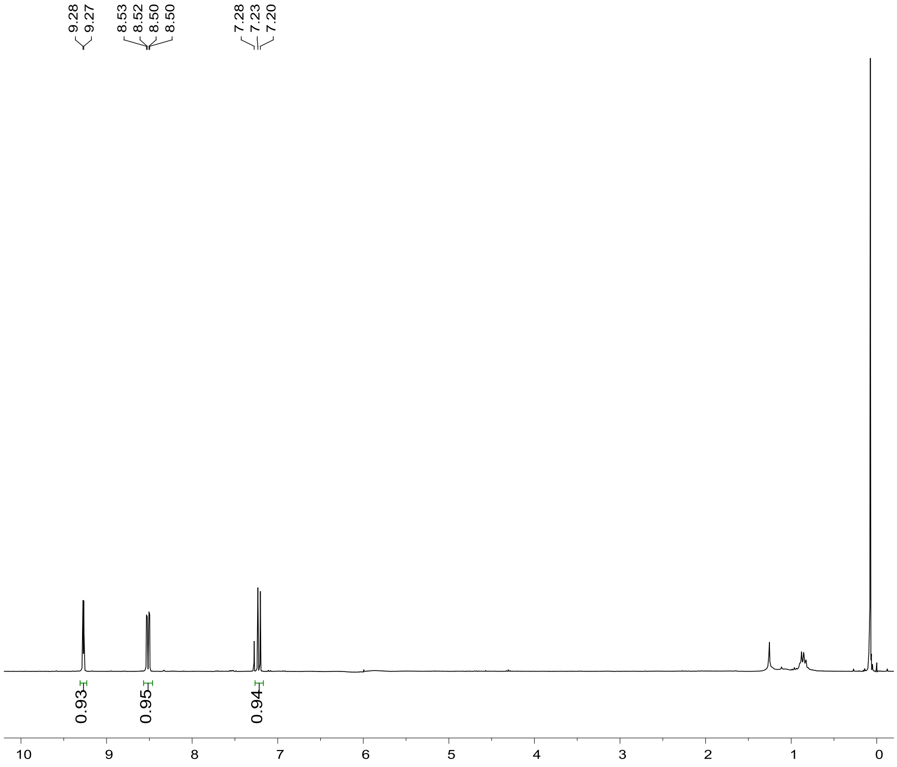

Parameter	Value
Title	zhanghao-H.35.fid
Comment	1H NMR of zh-NO2pyNCS
Origin	UXNMR, Bruker Analytische Messtechnik GmbH
Owner	root
Site	
Spectrometer	dpx300
Author	
Solvent	CDCl3
Temperature	23.2
Pulse Sequence	zg30
Experiment	1D
Probe	5 mm PABBO BB-1H Z-GRD Z862701/ 0035
Number of Scans	1
Receiver Gain	64
Relaxation Delay	2.0000
Pulse Width	10.7000
Acquisition Time	5.4658
Acquisition Date	2013-11-28T13:18:00
Modification Date	2013-11-28T13:18:21
Class	
Spectrometer Frequency	300.13
Spectral Width	5995.2
Lowest Frequency	-1198.6
Nucleus	1H
Acquired Size	32768
Spectral Size	65536

Figure S30. ^{13}C-NMR spectra of compound **4o**.

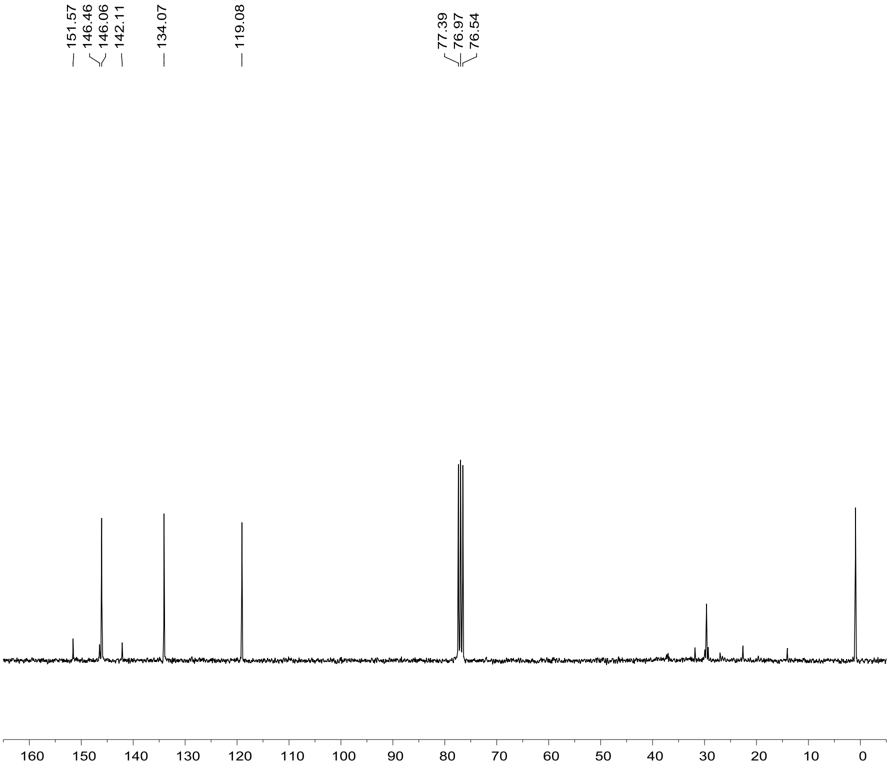

Parameter	Value
Title	zhanghao-13C.16.fid
Comment	13C NMR of zh-NO2PyNCS
Origin	UXNMR, Bruker Analytische Messtechnik GmbH
Owner	root
Site	
Spectrometer	dpx300
Author	
Solvent	CDCl3
Temperature	24.2
Pulse Sequence	zgdc30
Experiment	1D
Probe	5 mm PABBO BB-1H Z-GRD Z862701/ 0035
Number of Scans	620
Receiver Gain	3072
Relaxation Delay	1.5000
Pulse Width	12.0000
Acquisition Time	1.4451
Acquisition Date	2013-11-29T09:40:00
Modification Date	2013-11-29T10:11:07
Class	
Spectrometer Frequency	75.48
Spectral Width	22675.7
Lowest Frequency	-3797.5
Nucleus	13C
Acquired Size	32768
Spectral Size	65536

Figure S31. ^{1}H-NMR spectra of compound **4p**.

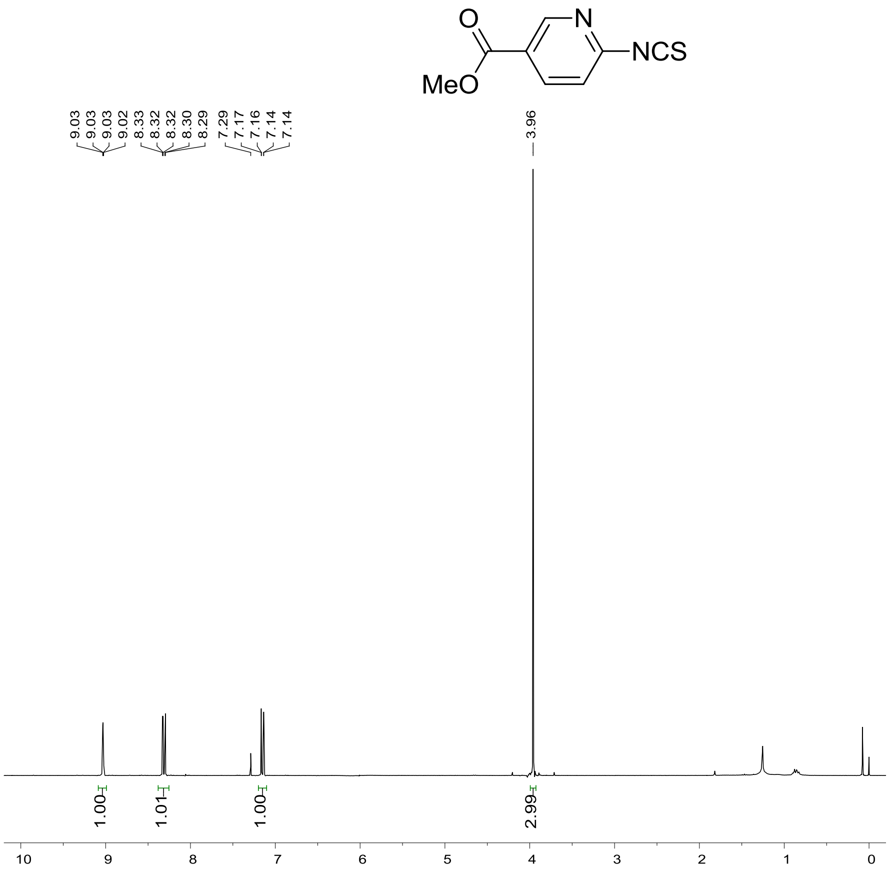

Parameter	Value
Title	zhanghao-H.29.fid
Comment	1H NMR of zh-coomeNCS
Origin	UXNMR, Bruker Analytische Messtechnik GmbH
Owner	root
Site	
Spectrometer	dpx300
Author	
Solvent	CDCl3
Temperature	24.0
Pulse Sequence	zg30
Experiment	1D
Probe	5 mm PABBO BB-1H Z-GRD Z862701/ 0035
Number of Scans	1
Receiver Gain	124
Relaxation Delay	2.0000
Pulse Width	10.7000
Acquisition Time	5.4658
Acquisition Date	2013-11-18T12:40:00
Modification Date	2013-11-18T12:40:16
Class	
Spectrometer Frequency	300.13
Spectral Width	5995.2
Lowest Frequency	-1194.2
Nucleus	1H
Acquired Size	32768
Spectral Size	65536

Figure S32. ^{13}C-NMR spectra of compound **4p**.

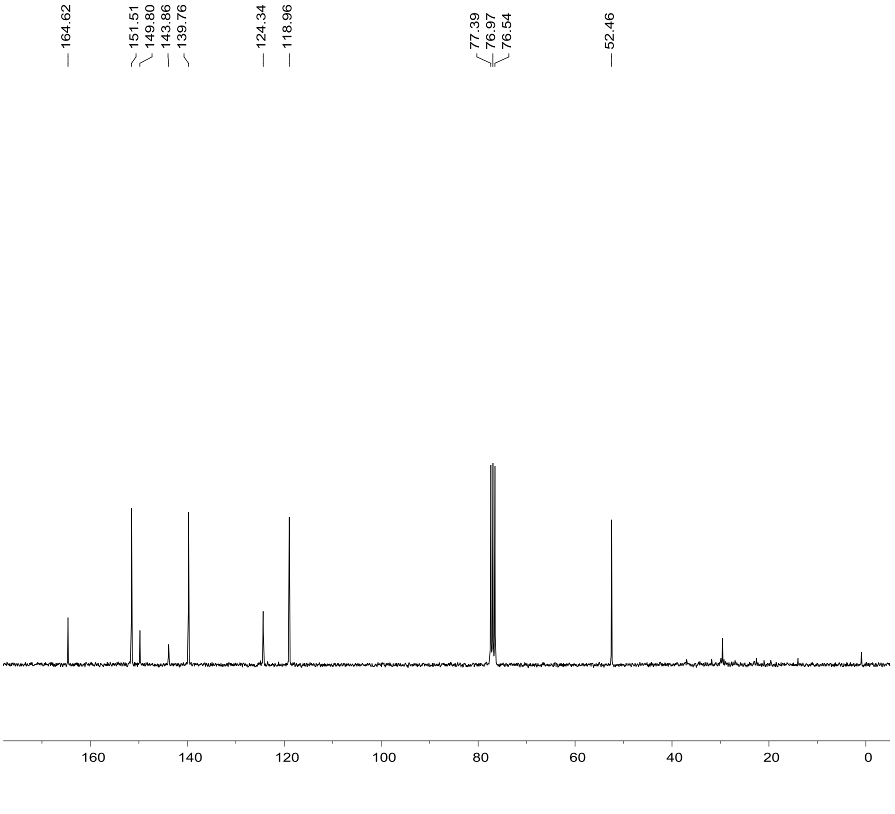

Parameter	Value
Title	zhanghao-13C.11.fid
Comment	13C NMR of zh-COOMeNCS
Origin	UXNMR, Bruker Analytische Messtechnik GmbH
Owner	root
Site	
Spectrometer	dpx300
Author	
Solvent	CDCl3
Temperature	24.0
Pulse Sequence	zgdc30
Experiment	1D
Probe	5 mm PABBO BB-1H Z-GRD Z862701/ 0035
Number of Scans	586
Receiver Gain	7168
Relaxation Delay	1.5000
Pulse Width	12.0000
Acquisition Time	1.4451
Acquisition Date	2013-11-18T19:38:00
Modification Date	2013-11-18T19:51:56
Class	
Spectrometer Frequency	75.48
Spectral Width	22675.7
Lowest Frequency	-3797.2
Nucleus	13C
Acquired Size	32768
Spectral Size	65536

Figure S33. ^{1}H-NMR spectra of compound **4q**.

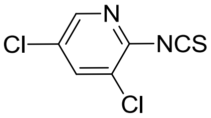

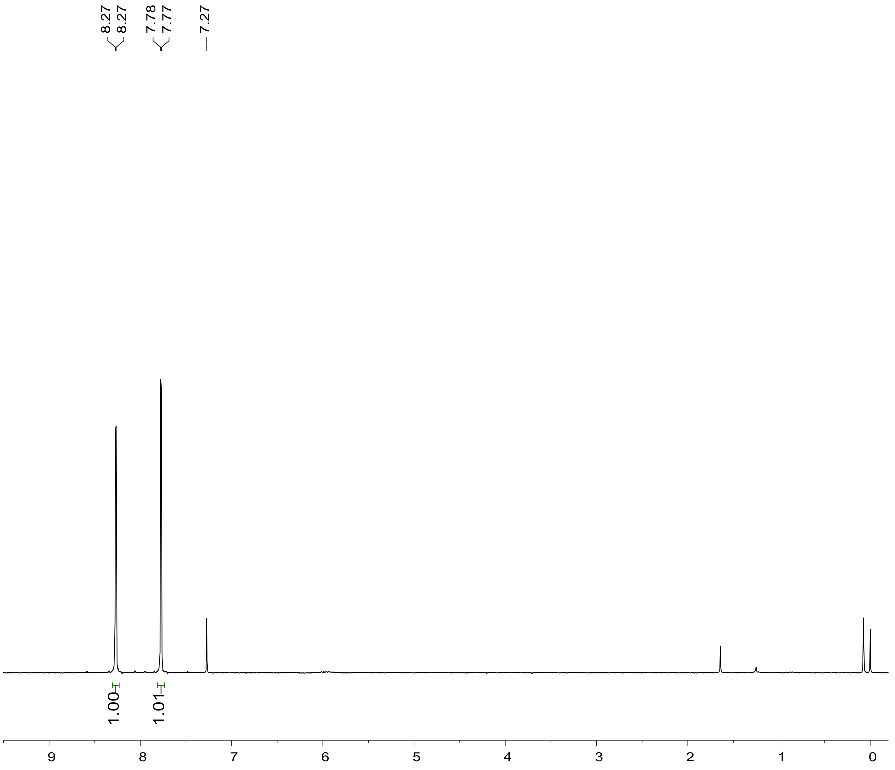

Parameter	Value
Title	zhanghao-H.20.fid
Comment	1H NMR of zh-2clNCS
Origin	UXNMR, Bruker Analytische Messtechnik GmbH
Owner	root
Site	
Spectrometer	dpx300
Author	
Solvent	DMSO
Temperature	0.0
Pulse Sequence	zg30
Experiment	1D
Probe	5 mm PABBO BB-1H Z-GRD Z862701/ 0035
Number of Scans	1
Receiver Gain	256
Relaxation Delay	2.0000
Pulse Width	10.7000
Acquisition Time	5.4658
Acquisition Date	2013-11-04T17:22:00
Modification Date	2013-11-04T17:22:39
Class	
Spectrometer Frequency	300.13
Spectral Width	5995.2
Lowest Frequency	-1199.8
Nucleus	1H
Acquired Size	32768
Spectral Size	65536

Figure S34. ^{13}C-NMR spectra of compound **4q**.

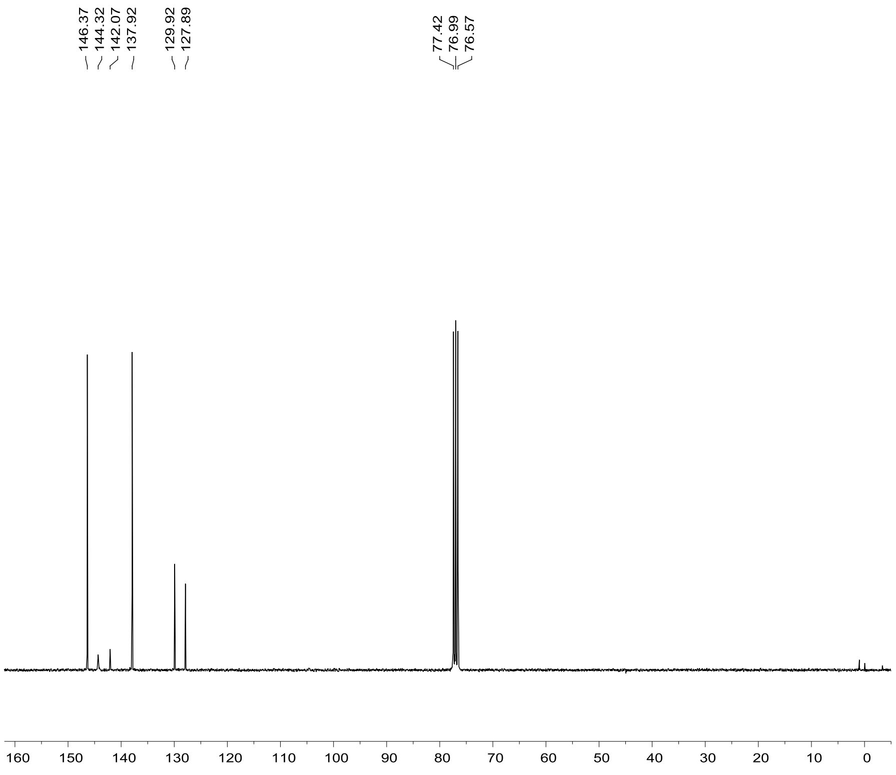

Parameter	Value
Title	zhanghao-13C.3.fid
Comment	13C NMR of zh-2ClNCS
Origin	UXNMR, Bruker Analytische Messtechnik GmbH
Owner	root
Site	
Spectrometer	dpx300
Author	
Solvent	CDCl3
Temperature	24.0
Pulse Sequence	zgdc30
Experiment	1D
Probe	5 mm PABBO BB-1H Z-GRD Z862701/ 0035
Number of Scans	4096
Receiver Gain	3072
Relaxation Delay	1.5000
Pulse Width	12.0000
Acquisition Time	1.4451
Acquisition Date	2013-11-04T19:22:00
Modification Date	2013-11-04T22:45:06
Class	
Spectrometer Frequency	75.48
Spectral Width	22675.7
Lowest Frequency	-3794.1
Nucleus	13C
Acquired Size	32768
Spectral Size	65536

Figure S35. ^{1}H-NMR spectra of compound **4r**.

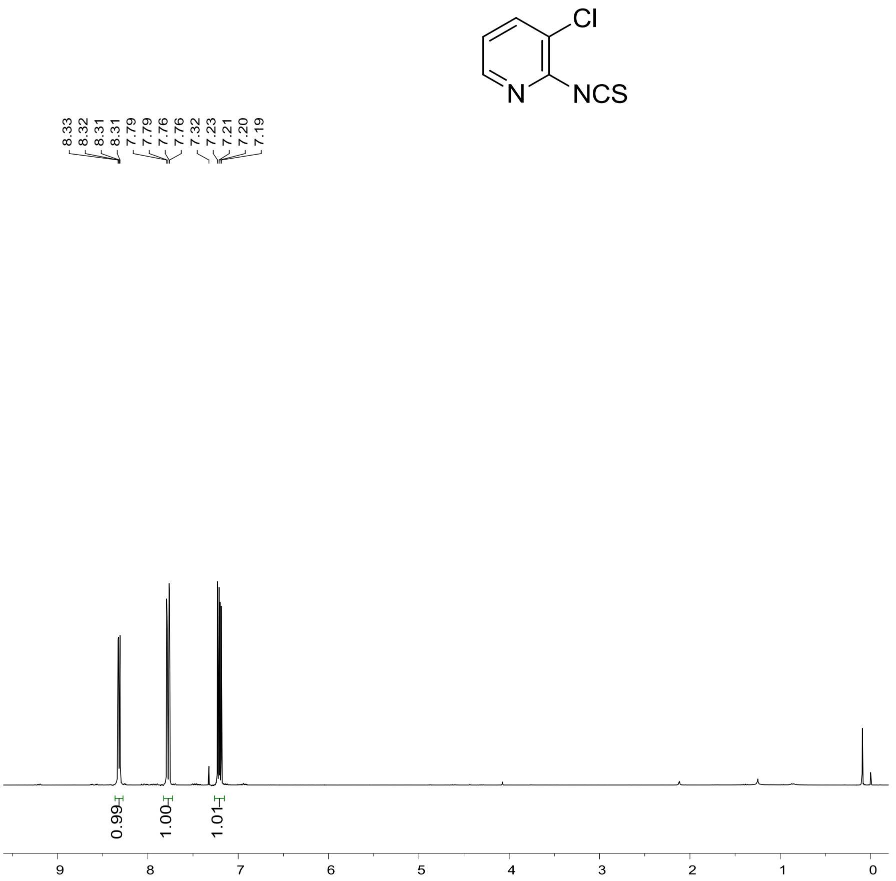

Parameter	Value
Title	zhanghao-H.37.fid
Comment	1H NMR of zh-3Cl2NCS
Origin	UXNMR, Bruker Analytische Messtechnik GmbH
Owner	root
Site	
Spectrometer	dpx300
Author	
Solvent	CDCl3
Temperature	24.2
Pulse Sequence	zg30
Experiment	1D
Probe	5 mm PABBO BB-1H Z-GRD Z862701/ 0035
Number of Scans	6
Receiver Gain	64
Relaxation Delay	2.0000
Pulse Width	10.7000
Acquisition Time	5.4658
Acquisition Date	2013-12-03T18:03:00
Modification Date	2013-12-03T18:03:54
Class	
Spectrometer Frequency	300.13
Spectral Width	5995.2
Lowest Frequency	-1183.9
Nucleus	1H
Acquired Size	32768
Spectral Size	65536

Figure S36. ^{13}C-NMR spectra of compound **4r**.

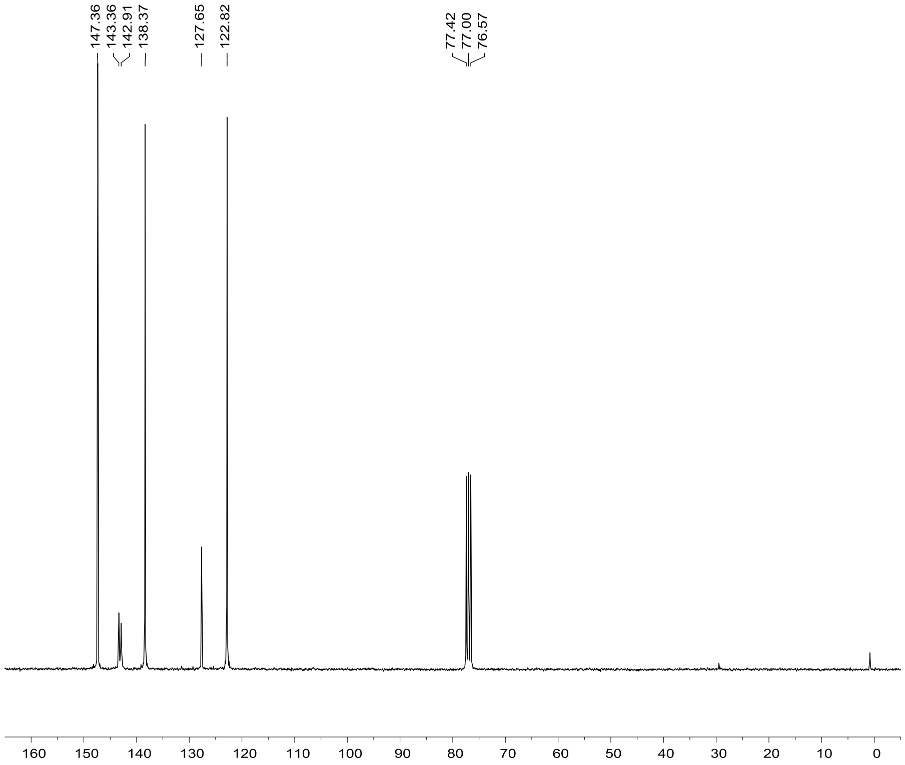

Parameter	Value
Title	zhanghao-13C.17.fid
Comment	13C NMR of zh-3Cl2NCS
Origin	UXNMR, Bruker Analytische Messtechnik GmbH
Owner	root
Site	
Spectrometer	dpx300
Author	
Solvent	CDCl3
Temperature	25.2
Pulse Sequence	zgdc30
Experiment	1D
Probe	5 mm PABBO BB-1H Z-GRD Z862701/ 0035
Number of Scans	2566
Receiver Gain	4096
Relaxation Delay	1.5000
Pulse Width	12.0000
Acquisition Time	1.4451
Acquisition Date	2013-12-03T18:12:00
Modification Date	2013-12-03T20:20:23
Class	
Spectrometer Frequency	75.48
Spectral Width	22675.7
Lowest Frequency	-3805.1
Nucleus	13C
Acquired Size	32768
Spectral Size	65536

Figure S37. ^{1}H-NMR spectra of compound **4s**.

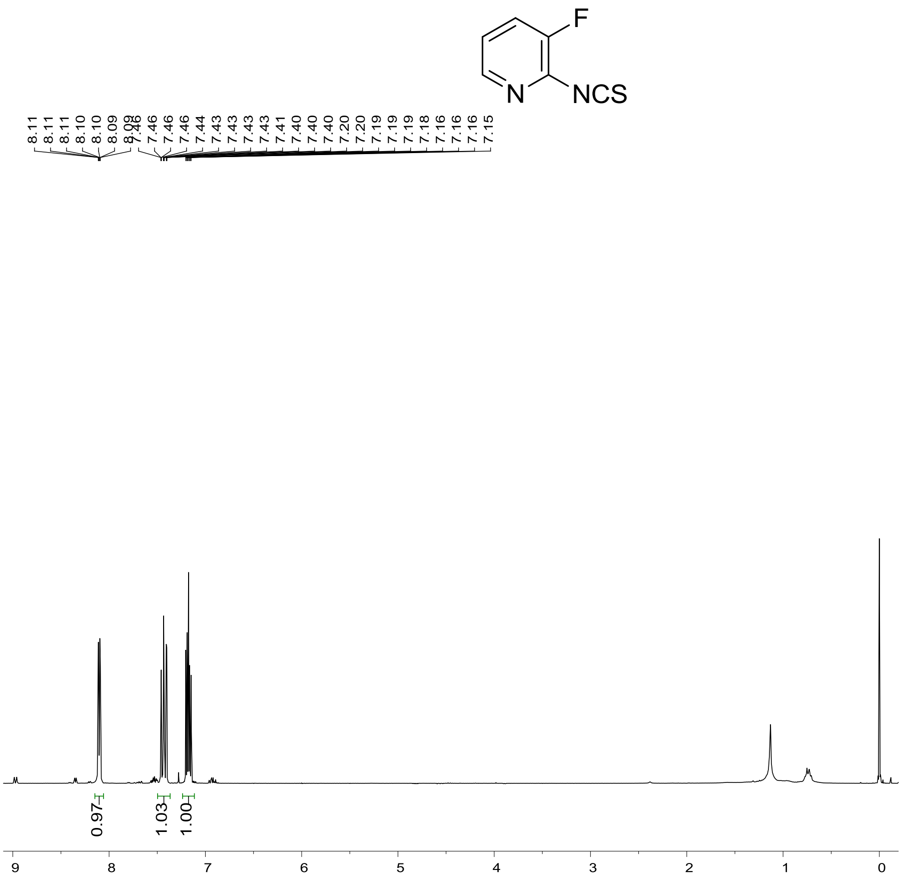

Parameter	Value
Title	zhanghao-H.38.fid
Comment	1H NMR of zh-3F2NCS
Origin	UXNMR, Bruker Analytische Messtechnik GmbH
Owner	root
Site	
Spectrometer	dpx300
Author	
Solvent	CDCl3
Temperature	25.2
Pulse Sequence	zg30
Experiment	1D
Probe	5 mm PABBO BB-1H Z-GRD Z862701/ 0035
Number of Scans	4
Receiver Gain	32
Relaxation Delay	2.0000
Pulse Width	10.7000
Acquisition Time	5.4658
Acquisition Date	2013-12-03T20:30:00
Modification Date	2013-12-03T20:30:26
Class	
Spectrometer Frequency	300.13
Spectral Width	5995.2
Lowest Frequency	-1197.5
Nucleus	1H
Acquired Size	32768
Spectral Size	65536

Figure S38. ^{13}C-NMR spectra of compound **4s**.

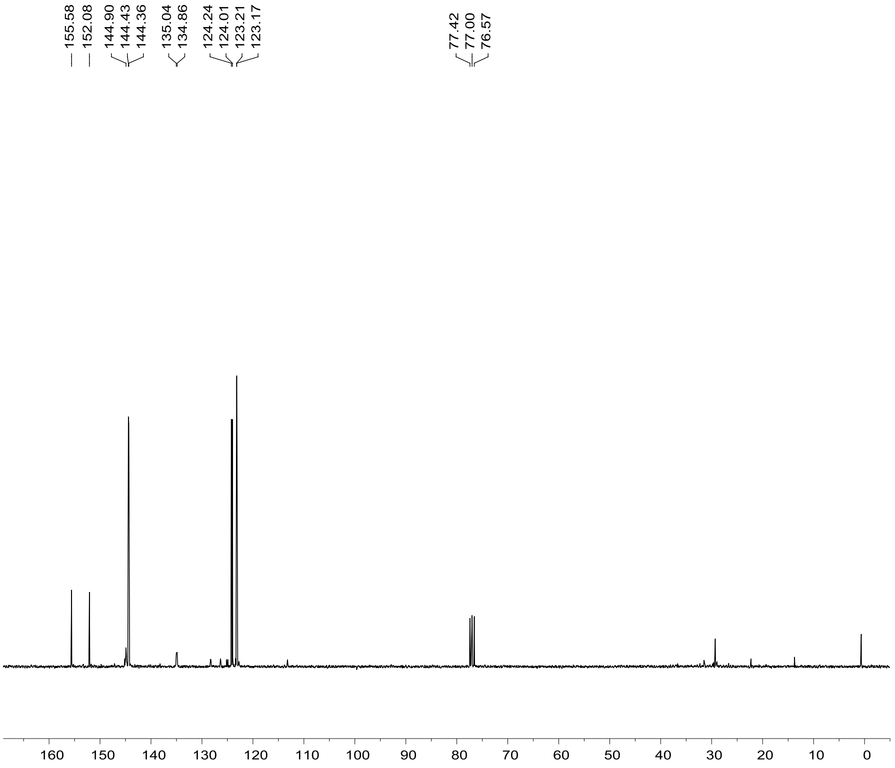

Parameter	Value
Title	zhanghao-13C.18.fid
Comment	13C NMR of zh-3F2NCS
Origin	UXNMR, Bruker Analytische Messtechnik GmbH
Owner	root
Site	
Spectrometer	dpx300
Author	
Solvent	CDCl3
Temperature	298.2
Pulse Sequence	zgdc30
Experiment	1D
Probe	5 mm PABBO BB-1H Z-GRD Z862701/ 0035
Number of Scans	202
Receiver Gain	2048
Relaxation Delay	1.5000
Pulse Width	12.0000
Acquisition Time	1.4451
Acquisition Date	2013-12-03T20:37:00
Modification Date	2013-12-03T20:45:53
Class	
Spectrometer Frequency	75.48
Spectral Width	22675.7
Lowest Frequency	-3816.9
Nucleus	13C
Acquired Size	32768
Spectral Size	65536

Figure S39. ^{1}H-NMR spectra of compound **4t**.

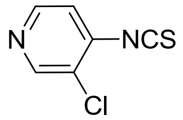

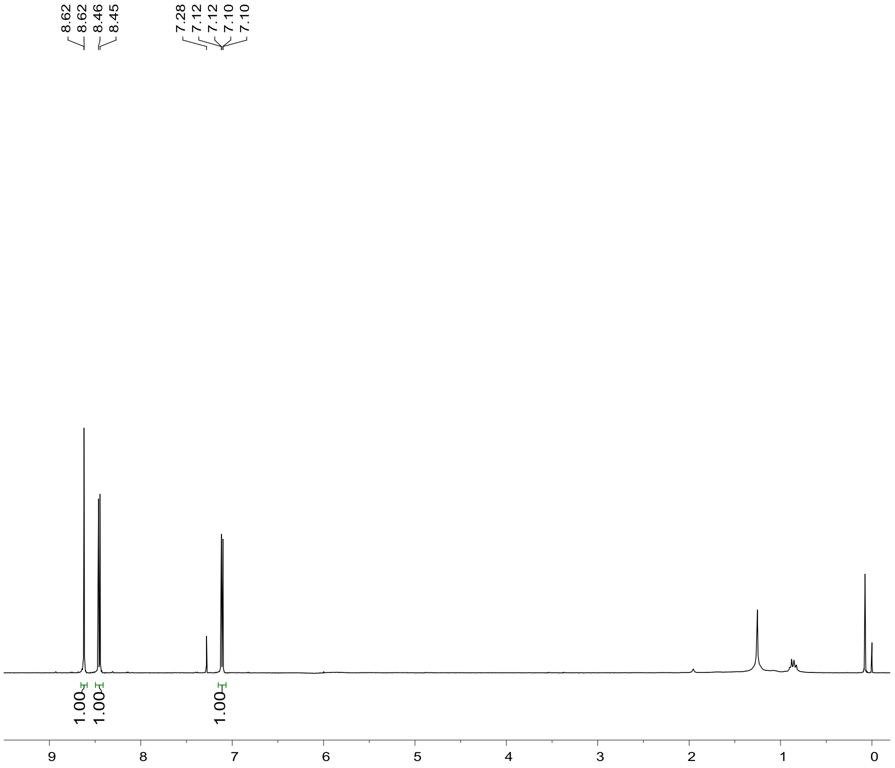

Parameter	Value
Title	zhanghao-H.31.fid
Comment	1H NMR of zh-4N3CLNCS
Origin	UXNMR, Bruker Analytische Messtechnik GmbH
Owner	root
Site	
Spectrometer	dpx300
Author	
Solvent	CDCl3
Temperature	24.0
Pulse Sequence	zg30
Experiment	1D
Probe	5 mm PABBO BB-1H Z-GRD Z862701/ 0035
Number of Scans	1
Receiver Gain	124
Relaxation Delay	2.0000
Pulse Width	10.7000
Acquisition Time	5.4658
Acquisition Date	2013-11-22T10:47:00
Modification Date	2013-11-22T10:47:07
Class	
Spectrometer Frequency	300.13
Spectral Width	5995.2
Lowest Frequency	-1196.9
Nucleus	1H
Acquired Size	32768
Spectral Size	65536

Figure S40. ^{13}C-NMR spectra of compound **4t**.

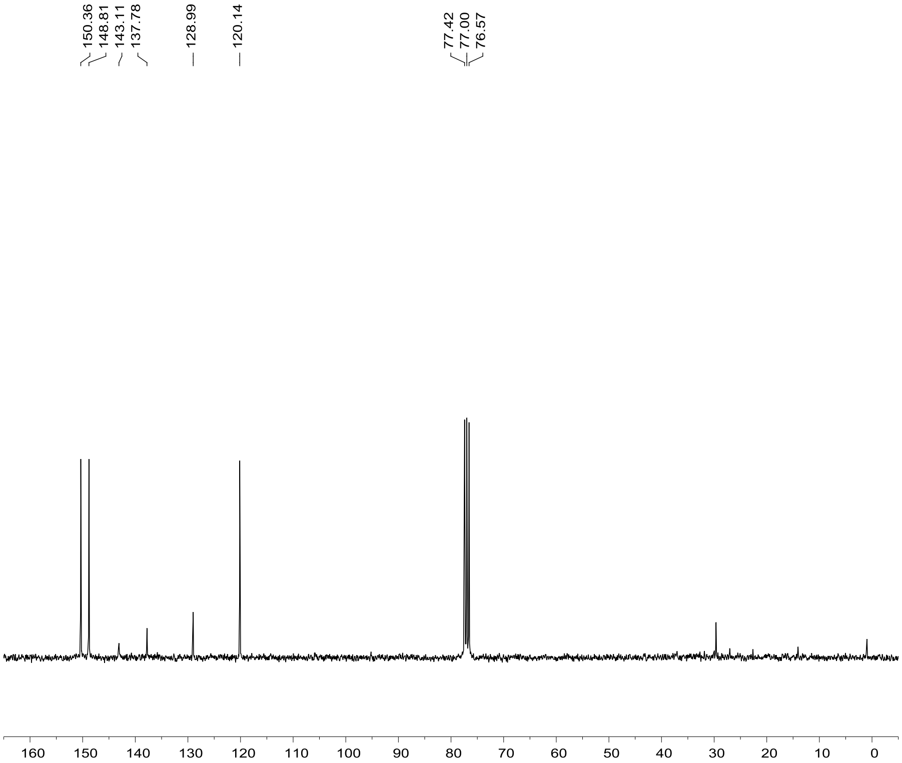

Parameter	Value
Title	zhanghao-13C.12.fid
Comment	13C NMR of zh-4N3ClNCS
Origin	UXNMR, Bruker Analytische Messtechnik GmbH
Owner	root
Site	
Spectrometer	dpx300
Author	
Solvent	CDCl3
Temperature	24.0
Pulse Sequence	zgdc30
Experiment	1D
Probe	5 mm PABBO BB-1H Z-GRD Z862701/ 0035
Number of Scans	310
Receiver Gain	9216
Relaxation Delay	1.5000
Pulse Width	12.0000
Acquisition Time	1.4451
Acquisition Date	2013-11-22T18:09:00
Modification Date	2013-11-22T18:24:09
Class	
Spectrometer Frequency	75.48
Spectral Width	22675.7
Lowest Frequency	-3794.8
Nucleus	13C
Acquired Size	32768
Spectral Size	65536
